# CMOS Scaling for the 5 nm Node and Beyond: Device, Process and Technology

**DOI:** 10.3390/nano14100837

**Published:** 2024-05-09

**Authors:** Henry H. Radamson, Yuanhao Miao, Ziwei Zhou, Zhenhua Wu, Zhenzhen Kong, Jianfeng Gao, Hong Yang, Yuhui Ren, Yongkui Zhang, Jiangliu Shi, Jinjuan Xiang, Hushan Cui, Bin Lu, Junjie Li, Jinbiao Liu, Hongxiao Lin, Haoqing Xu, Mengfan Li, Jiaji Cao, Chuangqi He, Xiangyan Duan, Xuewei Zhao, Jiale Su, Yong Du, Jiahan Yu, Yuanyuan Wu, Miao Jiang, Di Liang, Ben Li, Yan Dong, Guilei Wang

**Affiliations:** 1 Research and Development Center of Optoelectronic Hybrid IC, Guangdong Greater Bay Area Institute of Integrated Circuit and System, Guangzhou 510535, China; zhouziwei@giics.com.cn (Z.Z.); renyuhui@giics.com.cn (Y.R.); linhongxiao@giics.com.cn (H.L.); caojiaji@giics.com.cn (J.C.); hechuangqi211@126.com (C.H.); xyduan93@163.com (X.D.); wuyuanyuan@giics.com.cn (Y.W.); liben@giics.com.cn (B.L.); 2 Key Laboratory of Microelectronics Devices & Integrated Technology, Institute of Microelectronics, Chinese Academy of Sciences, Beijing 100029, China; wuzhenhua@ime.ac.cn (Z.W.); kongzhenzhen@ime.ac.cn (Z.K.); gaojianfeng@ime.ac.cn (J.G.); yanghong@ime.ac.cn (H.Y.); zhangyongkui@ime.ac.cn (Y.Z.); lijunjie@ime.ac.cn (J.L.); liujinbiao@ime.ac.cn (J.L.); limengfan69@163.com (M.L.); zhaoxuewei@ime.ac.cn (X.Z.); sujiale@ime.ac.cn (J.S.); duyong@ime.ac.cn (Y.D.); yujiahan@ime.ac.cn (J.Y.); dongyan2019@ime.ac.cn (Y.D.); 3 Beijing Superstring Academy of Memory Technology, Beijing 100176, China; jiangliu.shi@bjsamt.org.cn (J.S.); jinjuan.xiang@bjsamt.org.cn (J.X.); miao.jiang@bjsamt.org.cn (M.J.); di.liang@bjsamt.org.cn (D.L.); 4 Jiangsu Leuven Instruments Co., Ltd., Xuzhou 221300, China; hushan.cui@gmail.com; 5 School of Physics and Information Engineering, Shanxi Normal University, Linfen 041004, China; lubinsxnu@sina.cn; 6 Institute of Microelectronics, University of Chinese Academy of Sciences, Beijing 100049, China; xuhaoqing@ime.ac.cn; 7 Hefei National Laboratory, University of Science and Technology of China, Hefei 230088, China

**Keywords:** CMOS, process integration, nanoscale transistors, FDSOI, GAA, TFET

## Abstract

After more than five decades, Moore’s Law for transistors is approaching the end of the international technology roadmap of semiconductors (ITRS). The fate of complementary metal oxide semiconductor (CMOS) architecture has become increasingly unknown. In this era, 3D transistors in the form of gate-all-around (GAA) transistors are being considered as an excellent solution to scaling down beyond the 5 nm technology node, which solves the difficulties of carrier transport in the channel region which are mainly rooted in short channel effects (SCEs). In parallel to Moore, during the last two decades, transistors with a fully depleted SOI (FDSOI) design have also been processed for low-power electronics. Among all the possible designs, there are also tunneling field-effect transistors (TFETs), which offer very low power consumption and decent electrical characteristics. This review article presents new transistor designs, along with the integration of electronics and photonics, simulation methods, and continuation of CMOS process technology to the 5 nm technology node and beyond. The content highlights the innovative methods, challenges, and difficulties in device processing and design, as well as how to apply suitable metrology techniques as a tool to find out the imperfections and lattice distortions, strain status, and composition in the device structures.

## 1. Introduction

The development of CMOS technology is reaching its final stage where the transistor structure appears to be the most complicated. After more than fifty years following Moore’s Law, the technology roadmap will finally be finishing in a few years. Simply, downscaling is approaching a brick wall where our technology is unable to continue developing beyond the 3 nm node anymore. The main issues are the uniformity, reproducibility, and quality of nanomaterials in mass production when the threshold has been reached. At that stage, where we are not able to pack more transistors in a lateral direction on the chip, we try to pack them in a vertical direction [1,2,3]. Then, the vertical transistors are designed with GAA in mind to create an innovative idea for tackling the SCEs. However, this design is very appealing, but creates a lot of difficulties such as creating contacts between metal and individual or stacks of transistors [4,5]. Therefore, other approaches, e.g., nanosheet (NS) transistors and fin field-effect transistors (FinFETs) which are fully depleted on SOI (FDSOI) wafers, have recently been proposed. In this way, the issue of SCEs is diminished; furthermore, we will still be able to scale down the transistors for some years to come. In the FDSOI technology, the issue of transistor SCEs is solved, but manufacturing the required SOI wafers appears to be a new challenge. The vertical scaling of SOI wafers will suffer from the non-uniformity of the Si cap and oxide box thickness. This means that it is not easy to manufacture SOI wafers to follow scaling-down procedures to reach a few nanometers (nm). A brilliant solution is to turn over the CMOS technology to TFETs where band-to-band tunneling (BTBT) requires significantly lower power consumption. Such types of chips will fulfil all the demands for low power consumption, which has been the in Moore’s technology roadmap for years. Although TFETs offer an excellent platform for the industry, the problem of the stability of tunneling effect is still unsolved [1,3].

This review article presents the process integrity of CMOS to 5 nm and beyond. The content includes a survey of articles with a focus on the path to the end of the technology roadmap. Many issues around device design (e.g., GAA and tunneling FET), process difficulties and challenges, testing and characterizations, and introducing new materials are presented. The novelty of this review article lies in presenting the critical analysis and insights of the CMOS technology in the past, present, and future. For better navigation, we also provide the following short table.
Part I: Nanoscale tranisitor designsThe designs of Nanoscale Transistors in Approach to the End of Technology Roadmap and Beyond MooreAdvanced TCAD with AI for the 5 nm node and beyondPart II: Process of Nanoscale FETsAdvanced Lithography TechniqueEpitaxy in Transistor StructuresImplantation and Advanced Doping MethodsHKMG, ALD Technique and Negative Capacitance MaterialsAdvanced Etchning for Nano-Transistor StructuresWet Etch and CleaningMetal Materials InterconnectAdvanced Devices ReliabilityPart III: Materials in Beyond Moore EraIII–V MaterialsSubstrate Engineering (GOI and GeSnOI)Beyond Moore Era-Si OptoelectronicsPart IV: Metrology technologyAdvanced Material and Structural Analysis of Miniaturized CMOS

**Part One: Nanoscale transistor designs**

## 2. The Designs of Nanoscale Transistors in Approach to the End of Technology Roadmap and beyond Moore

### 2.1. Novel Structure, Gate-All-Around FETs

Compared with the Lateral GAA devices, Vertical GAA devices have more integration freedom in the vertical direction, which can increase the design space of gates, sources, and drains, reduce the area occupied by devices, and make it easier to realize vertical stacking of multi-layer devices. The wiring method further increases the integration density; therefore, it has become a basic device with great potential in logic and memory chip manufacturing technologies such as CMOS and high-density DRAM at 2 nanometers and below. Due to the space limitation and abovementioned advantages of Vertical GAA devices, we only focus on the Vertical GAA devices here. In our previous review article [1], we have talked about vertical GAA FET (VGAAFET), which is selected as one of the candidate transistors for new CMOS technology in the future [2,3]. As the channel is orthogonal to the wafer plane, VGAAFETs have the advantages of relieving Contacted Gate Pitch (CGP) constraints against further scaling [4], reducing the area of standard cells [5], and increasing integration density [6].

**Figure 1:**
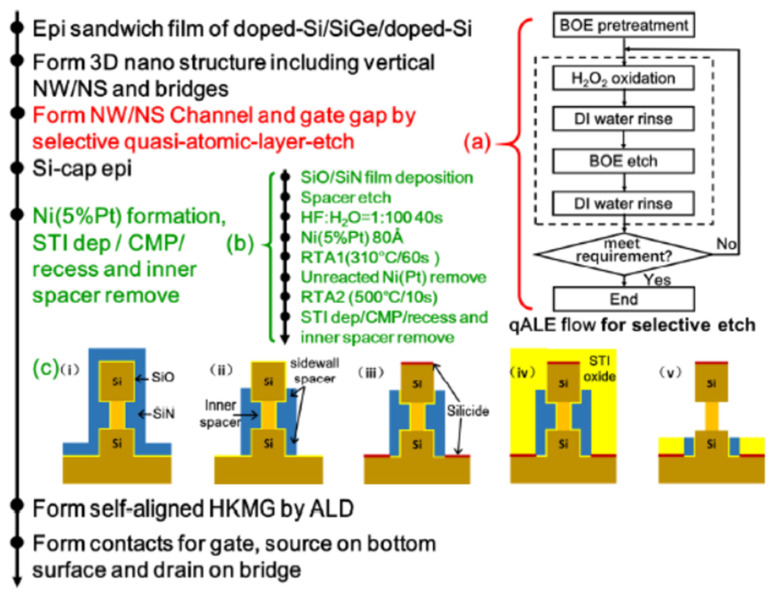
The process of VSAFETs, including the flow of selective qALE, and the flow and diagram of the Ni (Pt) silicide process. Reprinted with permission from ref. [9]. Copyright 2021 IEEE Publisher.

**Figure 2:**
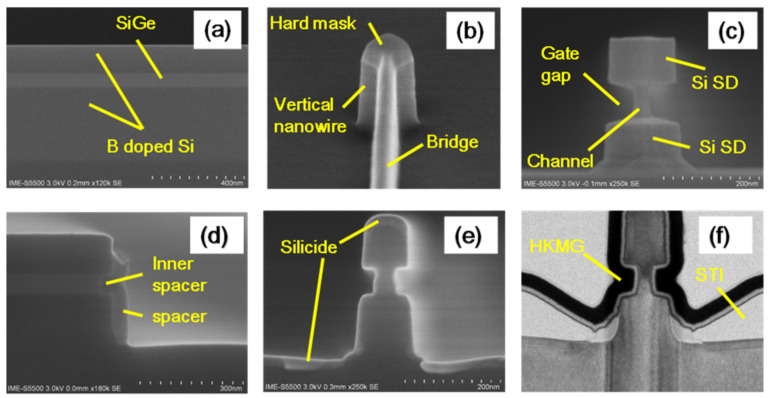
SEM micrographs: (**a**) epitaxially grown Si/SiGe/Si multilayers, (**b**) the tilted image of 3D nanostructure, (**c**) after qALE, (**d**) after inner spacer and sidewall spacer formation, (**e**) after silicide formation in structure wafer, and (**f**) TEM image after HKMG process. Reprinted with permission from ref. [9]. Copyright 2021 IEEE Publisher.

**Figure 3:**
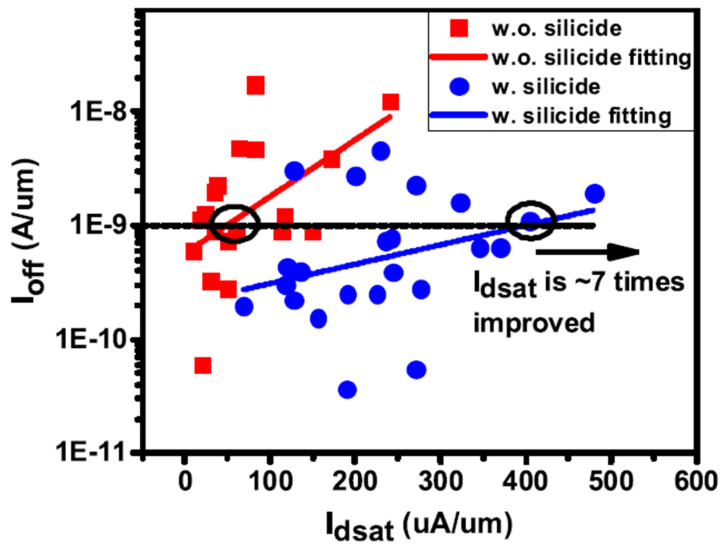
I_off_–I_dsat_ data from transistors for with and without silicided pVSAFETs. Reprinted with permission from ref. [9]. Copyright 2021 IEEE Publisher.

As the variation in gate length is a key processing issue for VGAAFET [7], a new p-type vertical sandwich, GAAFET (pVSAFET), has been processed, where high-k metal gates (HKMGs) are self-aligned with little effective gate length variation [8,9]. Such VSAFETs were manufactured through Si/SiGe/Si stacked structure epitaxy, an isotropic quasi atomic layer etching (qALE) process, and gate replacement process, as shown in Figure 1a–c. The main advantages of VSAFETs are that the gate length of the devices is mainly determined by the SiGe thickness, with little deviation in the process, and the diameter of NW and thickness of NS are precisely controlled by the Si-selective isotropic qALE of SiGe. Following the process shown in Figure 1b, Ni (Pt) silicide was formed on the surface of the source and drain region of the device (Figure 2e), and the channel of the device was protected by the inner spacer shown in Figure 2d. The external resistance of devices with silicide is about four times slower than that without silicide, and I_dsat_ of with silicide is approximately seven times higher than that without silicide when I_OFF_ = 10^−9^ A/μm, as Figure 3 shows. The performance of VSAFET is sensitive to the thermal budget process, as a high thermal budget will cause doped boron to diffuse from the source and drain to the channel, resulting in poor electrical behavior. In addition, the threshold voltage can be tuned by adjusting the Ge component in the channel, Si cap film, and different work function layers.

Since the transistors structures are complicated, the integrity of the process flow has proved to be stepwise. The best choice of method is cross-section scanning electron microscopy (SEM) or transmission electron microscopy (TEM). Since many labs take a test sample along the process line, high-resolution scanning electron microscopy (HRSEM) is frequently used in line. Figure 2 demonstrates the most critical steps in VGAA transistors. The following points are the focus: (a,b) the quality of SiGe/Si in terms of defect density and interfacial roughness; (c) the shape and dimensions of the formed channel after etching; (d) spacer thickness; (e) lack of void creation after silicide formation; (f) the uniformity of high k deposition. 

**Figure 4:**
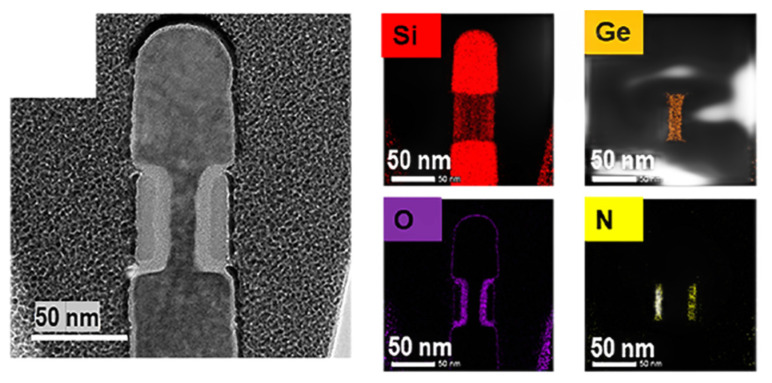
High-resolution TEM micrograph with detailed information about energy-dispersive X-ray spectroscopy (EDX) analysis. The SiN dummy gate has been dry-etched followed by dHF for 60 s to stripe the oxide layer on the sidewall. Reprinted from ref. [11]. Copyright 2021 ACS Publisher.

Because of the risk from n-type dopant segregation [10], n-type doping in the S/D of VSAFETs is preferably performed via the implantation process [11,12], which is different from the S/D in situ epitaxial doping process of pVSAFET. To protect the vertical channel during the S/D implantation, a dummy nitride is introduced in the gate (Fig.4). The n-type VSAFET is manufactured with CMOS-compatible technology and exhibits excellent output characteristics, including an excellent short-channel control capability (DIBL = 14 mV), extremely low leakage current, a small sub threshold swing of 67 mV/dec, and a high switching ratio (I_ON_/I_OFF_ = 3.5 × 10^6^). Thus, it could be an important candidate device for high-density and high-performance 3D-integrated circuits in the future (Figure 4).

**Figure 5:**
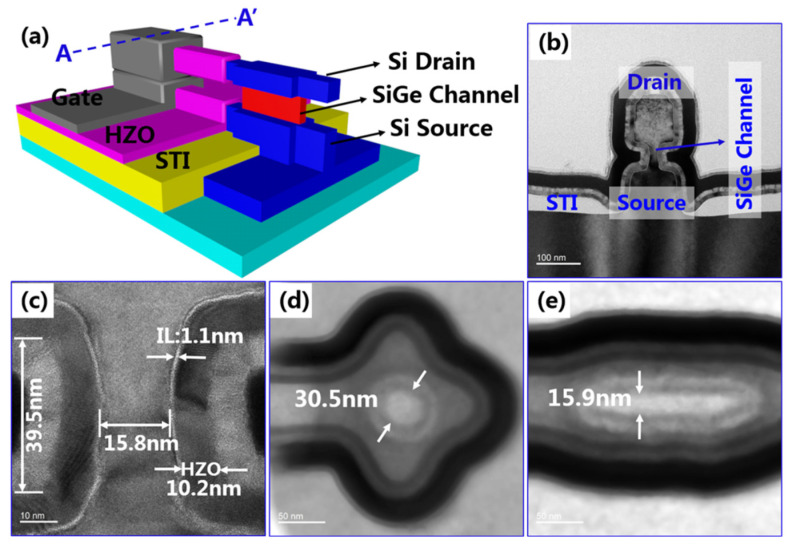
(**a**) A schematic of an Fe-VSAFET, (**b**) STEM cross-section for Fe-VSAFET across AA′ direction, (**c**) STEM cross-section of channel region, (**d**) top view of STEM of a nanowire device, and (**e**) top view of a nanosheet device. Reprinted with permission from ref. [13]. Copyright 2021 IEEE Publisher.

The vertical transistors have the advantages of nonvolatile data storage, nanosecond programming/erasing speed, low-power operation, ultra-long data storage time, and compatibility with the CMOS process. The ferroelectric field-effect transistor (FeFET) is considered as a candidate device for nonvolatile memory applications in the future. Due to the constraints of thick ferroelectric film (for example, ~10 nm Hf_x_Zr_1−x_O_2_), VGAAFET is more suitable for FeFET than FinFET and LGAAFET at the technology node of 5 nm and beyond. Figure 5 shows the ferroelectric VSAFETs (Fe-VSAFETs) with a self-aligned metal gate [13]. Fe-VSAFETs have excellent electrical characteristics such as I_ON_/I_OFF_ ratios of more than 10^7^, leakage currents of less than 1pA, long retention times, program/erase times of around 100 ns, and the largest memory window (MW) at about 2.3 V.

Further studies on GAAFET have been reported, including high-mobility channel materials, e.g., Ge/GeSn [14,15,16,17] and III–V [18], and wide bandgap materials, e.g., GaN [19,20]. 

### 2.2. Tunneling Field-Effect Transistor (TFET) Approach

As the size of MOSFET is scaled down to nanometers, the SCEs and power consumption become increasingly important issues for chips. In order to reduce the power consumption, the V_T_ and operation voltage (V_DD_) have to be reduced. However, for transistors with a constant sub-threshold swing (SS), reducing V_T_ and V_DD_ would lead to exponentially increased leakage current and static power consumption. Therefore, a smaller SS, namely extremely steep switch characteristic, is required. Unfortunately, due to the tail of the Boltzmann distribution, the SS of MOSFETs has a limitation of 60 mV/dec at room temperature. 

TFET operation is based on the BTBT mechanism. This can break the 60 mV/dec limitation of SS and allow further scaling of V_T_ and V_DD_ even with a reduced leakage current; therefore, this can be widely considered as the most promising candidate for future low-power applications.

In 2004, a carbon nanotube TFET with sub-60 mV/dec SS was reported [21]; afterwards, plenty of experimental studies were carried out for both nTFETs and pTFETs [22]. Some experimental reports based on Si and Ge materials are summarized in Table 1 [23]. It is clearly observed that most devices can exhibit a minimum SS smaller than 60 mV/dec, owing to the mature fabrication technology and nearly perfect semiconductor/oxide interface. However, an SS smaller than 60 mV/dec can only appear across a very small current range and at a very small drain current. Furthermore, the on-state current (I_ON_) is only around a few μA/μm, which results from the low BTBT probability owing to the large and indirect bandgap of Si and Ge. To boost the I_ON_, more attention is focused on the III–V material with a small bandgap and tunneling mass, and the hetero tunneling junction with a small tunneling barrier. Table 2 summarizes some works based on III–V materials [22,24,25,26,27,28,29,30,31,32,33,34,35,36,37]. It is clearly observed that I_ON_ levels are greatly improved due to the reduced tunneling barrier compared with those of conventional Si and Ge TFETs. However, it seems that it is harder for III–V TFETs to obtain an SS smaller than 60 mV/dec and some of them even show a minimum SS larger than 100 mV/dec, which is obviously not what we are expecting. Two factors may contribute to this deviation. One factor is the complex and poor stability of the interface between III–V and gate oxide inducing a high density of states, which causes Fermi pinning and degrades the gate control efficiency. The other factor is the lattice mismatch at the hetero tunneling junction inducing a large density of traps. This further leads to the trap-assisted-tunneling (TAT) process at the tunneling junction, which is nearly not under the control of gate bias. Therefore, the III–V TFETs usually exhibit a large leakage current and degraded SS.

In addition to the Si, Ge, and III–V materials, in recent years, some other new materials are also applied to improve TFET performance, such as a ferroelectric layer [55], WSe_2_/SnSe_2_ [56,57], graphene [58,59], MoS_2_/HfS_2_ [60], and WTe_2_/WS_2_ [61]. These are all excellent examples; however, the above problems are not totally addressed. Both high I_ON_ and low SS levels are required for the practical applications of TFETs; thus, further investigations are still needed to improve the device performance.

### 2.3. FDSOI Technology

The miniaturization of transistor dimensions has created faster chips with less power consumption [1,3]. However, as the chip size approaching its physical limit in recent years, SCEs has dominantly degraded the performance. Therefore, new structures are developed to overcome the physical limitations and further develop chip integration. At nodes below 28 nm, the traditional planar MOSFET structure has been basically abandoned, and multi-gate device structures with stronger gate controllability, e.g., FinFET, FDSOI, and GAA, have been proposed [2,3]. Due to their superior electrostatic control performance, FinFET and FDSOI technologies have overthrown the traditional planar body transistors. Although the cost of the FDSOI wafer is higher, it requires only small changes in production compared with the traditional bulk, allowing more of the existing technologies to be used. GAA is further improved based on FinFET, and the process cost will be higher. Based on the advantages of the merger of SOI and FinFET, as well as strain engineering, a better platform can be obtained in the technology roadmap, e.g., SOI- or strained SOI-FinFETs. It should be noted that FinFET used on bulk Si actually had many problems after reaching 5 nm. For example, its ever-increasing ratio of depth to width will make it difficult for the fins to maintain an upright shape under the internal stress of the material itself; as the gate width further increases with miniaturization, it will be difficult to fill multiple fin lines in one unit as in the past. Moreover, the static electricity problem of fin field-effect transistors will also seriously restrict the further improvement of transistor performance. Tinkering with FinFETs will eventually be insufficient, and new architectures are ready to emerge. Therefore, the use of FinFET architecture on FDSOI wafers is enough for the 5 nm node.

#### 2.3.1. What Is FDSOI (Architectures and Characteristics)

**Figure 6:**
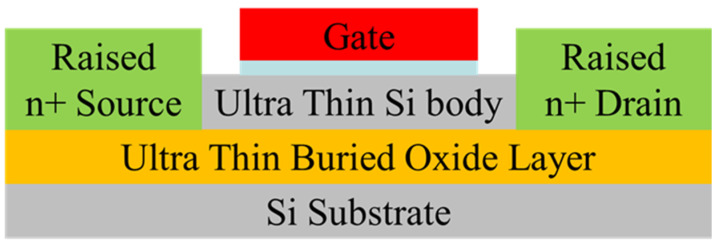
Schematic of planar FDSOI transistor.

**Figure 7:**
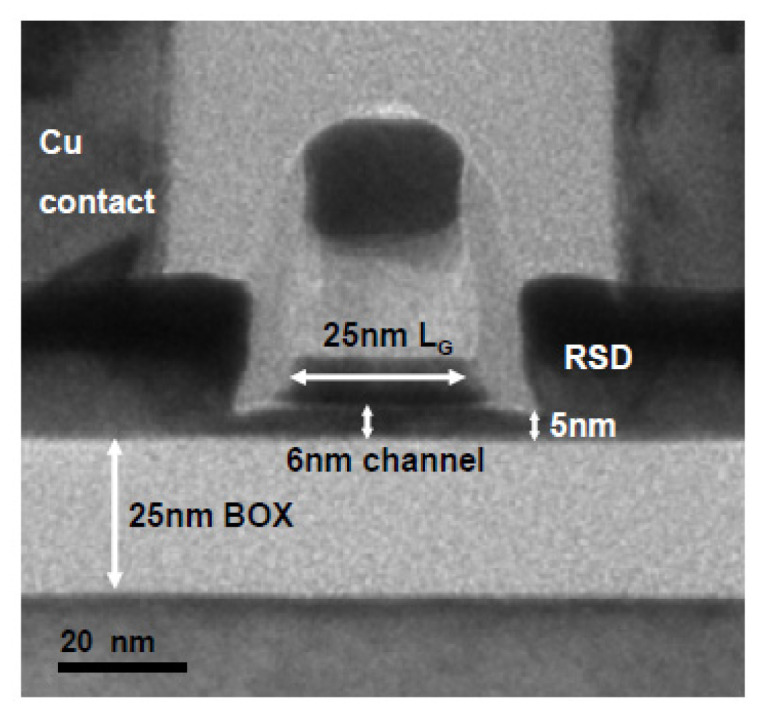
The typical cross-section TEM of an ultra-thin FDSOI device [66]. Reprinted with permission from ref. [66]. Copyright 2011 IOP Publishing.

The key difference between SOI MOS and a conventional MOS structure is the buried oxide layer (BOX) of SOI, which separates the transistor from the substrate. As shown in Figure 6, the thin transistor channel is fully depleted during operation and sandwiched between two insulating layers. Usually, the channel thickness is around 1/4–1/3 of the gate length. To avoid the large access series resistance caused by the direct growth of metal silicide, the structure of the raised source/drain (RSD) is generally adopted [62,63,64,65], as is shown in Figure 7. 

#### 2.3.2. FDSOI Salient Characteristics

**Figure 8:**
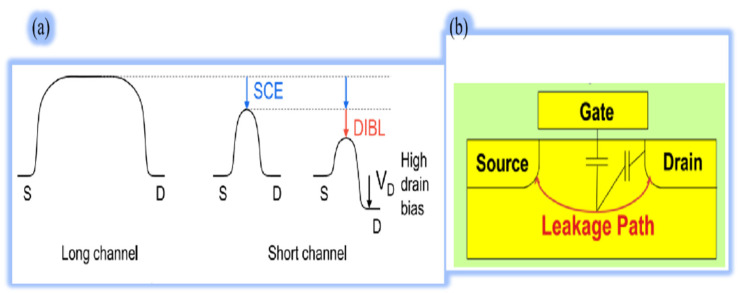
(**a**) Top of the band schematic, (**b**) bulk CMOS currents leakage path. Reprinted with permission from ref. [68]. Copyright 2012 IEEE Publisher.

When scaling down further, the SCEs and drain-induced barrier lowering (DIBL) induce the channel current uncontrolled by the gate as the shorter the gate length, the lower the barrier, as shown in Figure 8 [67,68]. DIBL is another SCE, which results from decreasing the channel barrier at higher drain voltage and lowers the V_T_. Thus, SCEs are intolerable while the channel length is less than the depletion width. In addition, other issues confining device performance also arise when scaling down, including a higher leakage current, higher SS [69], lower on-to-off current ratio, and random dopant fluctuation (RDF) [70,71]. 

FDSOI technology features improved the electrostatic controllability and channel carrier mobility of the gate, along with a decreased RDF. This technology possesses salient characteristics, such as a lower RDF with an undoped channel, eminent control of SCEs, higher carrier mobility, compatibility with planar processing library [72], lower mismatch variation, ultra-low-power applications [73,74,75], multi-V_T_ option [76], and a good back gate biasing option, among others. All these features make FDSOI technology the best power/performance/cost tradeoff choice. Near-zero V_T_ can be readily achieved in FDSOI without degrading other characteristics of the device [77], providing a great opportunity for ultra-low operating voltage (V_DD_) application, especially for primary RF and high-precision analog. 

It is demonstrated that ample control of SCEs achieved in FDSOI scaling down to 10 nm without performance degradation [78,79], benefiting from the inherent thin SOI architecture [80] and based on the investigation on SCEs [81], indicated that devices are still indomitable against SCEs even with a fairly thin SOI film [82]. Naturally, the leakage current into the substrate and gate-induced drain leakage (GIDL) current are largely lessened [83,84] thanks to the carriers being strictly confined within the thin channel. 

**Figure 9:**
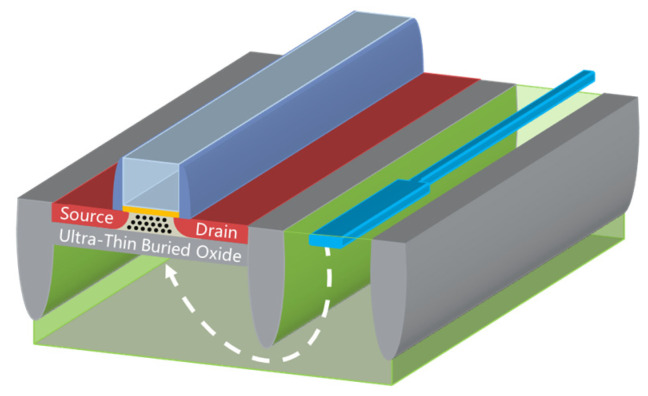
Schematic of body biasing in FDSOI.

Furthermore, with the implementation of back gate biasing, excellent SCE management was obtained [85,86], and electrostatic control represented a faster transition between off-state and on-state with decreased DIBL, SS, and variability. Utilizing various polarities [87,88,89] in conjunction with applying a bias to the well (Figure 9) [90,91,92], FDSOI V_T_ can be configured in different ways, enabling us to dynamically modulate the transistor speed [76,93], which is incomparably intrinsic to bulk CMOS or FinFET. Unlike the well in bulk CMOS PFET and NFET only corresponding with an n-type and p-type implant, respectively [94], the well in FDSOI features selectivity decided by the V_T_ required, meaning that the good polarity of the dopant is the same in the case of S/D [95]. Body bias is the most critical characteristic of FDSOI, consisting of forward body biasing (FBB) and reverse body biasing (RBB) [96]. By that, FDSOI possesses lower V_DD_ to achieve an identical drive current which leads to a decrease in both off- and on-state power, which profits the application of ultra-low power [97,98]. 

#### 2.3.3. FDSOI Roadmap and Future Perspectives

Recently, 28 nm and 22 nm node FDSOI technologies have been placed into a wide range of consumer products, especially low-power consumption and the high energy-efficiency market, thanks to the implementation of the ultra-thin BOX and SOI thickness techniques, which also enable nodes below 14, 10, and even sub-10 nm [80,99,100,101,102]. For the 7 nm node examined in the research, the SOI thickness could be thinned down to 4.5 nm [103].

Additionally, some enhancement methods to booster device performance, such as strain, new materials for the channel, access to contact, and spacers, have been recommended. With applications ranging from induced strain engineering [104] to the finite thin SOI layer of FDSOI [105], the general use of channel strain materials (intrinsically strained) is a very effective method [106,107]. Typical strained channel materials are biaxial tensile strain Si or biaxial compressive strain Ge on the SiGe layers, or uniaxial stress induced by the SiGe source/drains layer. Nitride liners are also commonly used to add stress to FETs [108,109]. However, strain integration in these low-dimensional channels is very challenging. An appealing approach to enhance the performance of such low-dimensional devices by boosting the carrier mobility is to replace the Si channel with a high-mobility material, such as Ge, SiGe, and III–V [110,111].

**Figure 10:**
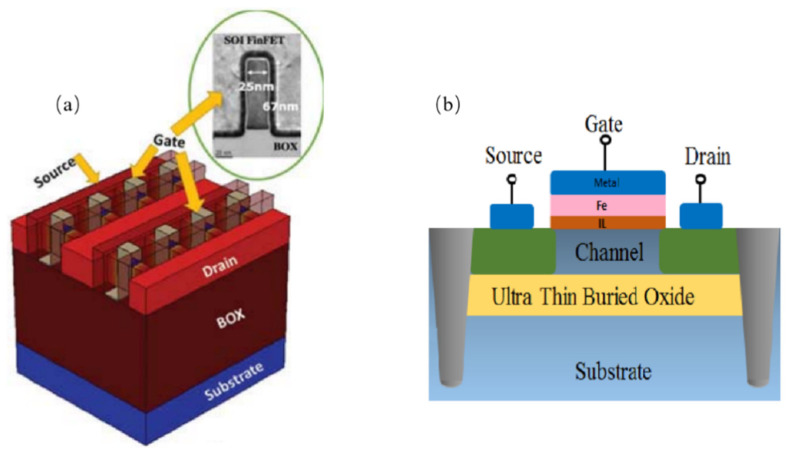
(**a**) New device structure for SOI FinFET [118] and (**b**) NC-FDSOI [119]. Reprinted with permission from ref. [118,119]. Copyright 2018 IEEE Publisher.

New MOS structures such as SOI/sSOI FinFET and negative capacitance FDSOI (NC-FDSOI) are introduced as follows (Figure 10). The more the gate numbers over the channel region are increased, the better their carrier transport control capability [112,113]. SOI/sSOI FinFET have an advantage over conventional single-gate devices concerning their performance in dealing with device leakage and current. Moreover, they have much lower process-induced variability due to SOI technology with BOX isolation instead of junction isolation [114]. 

In addition, negative capacitance field-effect transistor (NCFET) incorporating a ferroelectric layer is a promising solution to device scaling down by reducing effective oxide thickness [115,116]. Since there is no reduction in the thickness of the physical gate oxide layer or the interface silicon dioxide layer, it enhances gate control without sacrificing gate leakage current or mobility. NC-FDSOI is reported to scale to a 2 nm node with high-performance applications, and meets the 100 nA/μm leakage requirement in the simulation level [117]. The technology also the advantage in that both the I_OFF_ and I_ON_ of NC-FDSOI can be effectively adjusted by the back-gate bias. 

## 3. Advanced TCAD with AI for the 5 nm Node and beyond 

One of the major influences of AI on semiconductor applications is the use of AI-enabled technology computer-aided design (TCAD) tools and methods to optimize chip performance, power consumption, and reliability. AI can help engineers finish the layout design, verification, synthesis, and simulation, as well as to explore novel architectures and semiconductor materials. AI can also provide more efficient and scalable designs of complex systems-on-chip (SoCs) that integrate multiple functions and domains. TCAD plays a critical role in the design and pathfinding of state-of-the-art semiconductor devices. However, as semiconductor devices scale down to the 5 nm technology node and beyond, quantum-physics-induced effects are inevitable, leading to a huge rise not only in TCAD theory and methodology complexity, but also in computational cost. To improve the predictability and the computational efficiency of TCAD simulations, there have been many studies on developing advanced physical models beyond drift-diffusion DD frameworks [120], as well as incorporating high-performance computing [121]. Alternatively, artificial intelligence (AI) technology, including machine learning (ML) and deep learning (DL) approaches, is introduced into TCAD and implemented into not only atomistic calculation but also device- and circuit-level simulation. 

### 3.1. Atomistic Calculation with AI

Density functional theory (DFT) and DFT-based ab initio TCAD are powerful tools for calculating and analyzing the properties of the material in nanoscale semiconductor devices, especially when the critical dimension of devices reaches atomistic scale, and the quantum effect is not negligible. However, DFT theory is quite computationally expensive, and a trade-off between accuracy and efficiency is required. Therefore, novel machine learning approaches are introduced when considering that the neural network (NN) shows good capability in capturing nonlinearity and predicting targets according to given samples. One example is to solve the Schrodinger equation and achieve almost exact solutions for molecules with up to 30 electrons, whose efficiency outperforms the state-of-the-art variational ansatzes with high accuracy [122]. Another is that the NN performs as a wave-function Ansatz for the many-electron system in ab initio calculation and predicts the dissociation process of the nitrogen molecule and hydrogen chain [123]. The neural network architecture could also be part of the calculation. This has been demonstrated in a recent study, in which the neurons act as the tight-binding (TB) matrix elements in the Hamiltonian parameterization of the TB model for energy band calculation [124]. From calculation methodology to case studies, several studies have been reported in which machine-learning-augmented DFT works efficiently with high accuracy in atomistic modeling for devices, including the prediction of atomic force in phase change memory [125], the calculation of potential energy surface in SiGe alloys [126], and the simulation of surface reconstruction of the Si (111)-(7 × 7) surface [127].

**Figure 11:**
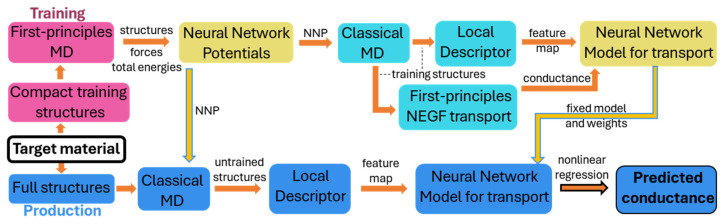
The proposed NN-NEGF computation framework.

In practical TCAD applications in the development of the 5 nm node and beyond, quantum transport features and device merits are essential in simulations. Apart from the calculation of electrostatic properties, the quantum transport simulation using the Non-Equilibrium Green Function (NEGF) method is performed along with the machine learning method as reported in recent studies. M. Burkle et al. have proposed an NN-NEGF simulation framework that could predict the conductance of a large system whose prediction accuracy agrees with the experiment qualitivity and the calculation cost is only a fraction of the conventional first-principle methods, as shown in Figure 11 [128]. Additionally, recently Y. Zhou et al. presented AD-NEGF, the first end-to-end differentiable NEGF model for quantum transport simulation, where the numerical calculation is carried out in the deep learning framework Pytorch v2.2.2, and the backward gradient is calculated efficiently using the proposed implicit layer technique [129]. As we could see above, the machine learning method has been a strong candidate to overcome the bottleneck of computation efficiency in the first-principle method for device analysis and optimization.

### 3.2. Semiclassical Device Simulation with AI

**Figure 12:**
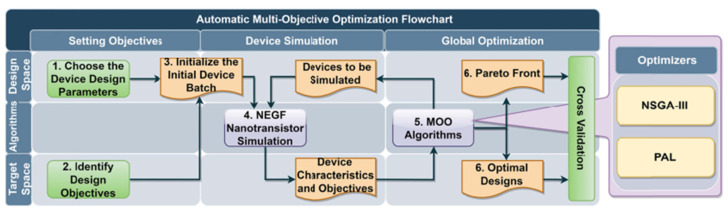
The proposed MOO framework couple with machine learning for 2D TMDC and black phosphorene FETs [139]. Reprinted with permission from ref. [139]. Copyright 2021 IEEE Publisher.

Although a lot of progress in atomistic simulation with AI has been made in recent years, the computational cost is still too expensive to satisfy the massive simulations demanded by the industry. Bridging TCAD and AI in the Drift-Diffusion framework, i.e., the semiclassical level, is more compatible to current sub-5 nm node development. To this end, the following updates have been proposed and addressed by several academic and industrial research groups. In general, traditional Design of Experiment (DOE) using TCAD is time-consuming due to a huge number of calculations. A possible remedy for this problem is machine learning-based modeling. It is commonly shown in recent studies that artificial neural networks (ANNs) have a remarkable ability in capturing nonlinear relationships with high accuracy between electrical characteristics and device parameters. This indicates a substantial decrease in computation cost in estimating electrostatic potential [130,131], capacitance–voltage (CV) relationship, current voltage (IV) [132,133,134], V_T_ [135], metal work function [136], as well as other figures of merit [137,138]. Furthermore, machine learning works well not only in the prediction of device characteristics but also in device optimization, where machine learning is coupled with a multi-objective optimization algorithm where the trade-off between electrical characteristics is carefully considered. For instance, a multi-objective optimization (MOO) framework is proposed with Pareto active learning to optimize 2D transition metal dichalcogenide (TMDC) and black phosphorene FETs as shown in Figure 12 [139]. Additionally, the 2D FET optimized by the proposed framework meets the International Roadmap of Devices and System target of 2025 and 2028 technology nodes.

### 3.3. Compact Modeling with AI

In addition to numerical simulations, analytical compact models for SPICE simulations are also very important. Compact modeling of emerging devices is essential for design-technology co-optimization (DTCO) and path-finding activities. However, quantum-physics-based standard models for FETs at the scaling limit which capture the non-linearity between electrical characteristics of transistors and design parameters demand a lot of time and domain expertise to be built [140,141]. In 2017, L. Zhang et al. proposed a new method that employs ANN design for the compact modeling of generic transistors [142], which is a novel option for the compact modeling of emerging devices. Detailed ANN design rules of compact modeling are discussed in ref. [143], where ANN model accuracy is optimized, and SPICE simulation turn-around time is reduced. Several models of novel devices, including ferroelectric FETs [144] and Si cold source GAA FETs [145], are reported to be built by ANN-based compact modeling, which indicates that ANN-based compact modeling is a promising candidate for DTCO and pathfinding.

**Part Two: Process of Nanoscale FETs**

## 4. Advanced Lithography Technique 

Rayleigh formula describes the resolution limit for optical lithography, shown as:

$$R=k1\frac{\lambda }{NA}$$

where *R* is the resolution term, NA stands for the numerical aperture of the project lens of the lithographic tool, λ denotes the wavelength of the applied light source, and the k1 factor is related to the challenges of the lithography.

The formula has clearly indicated the methods whereby the resolution can be improved.

(1) Wavelength reduction: i-line (365 nm), DUV KrF (248 nm), DUV ArF (193 nm), and EUV (13.5 nm).
(2) DUV NA increase: from 0.93 of ArF dry tool to 1.35 of ArF immersion at 193 nm lithographic tools.
(3) EUV NA increase: from 0.33 to 0.5 at current EUV tool and future high-NA EUV tools.
(4) K1 reduction through various patterning enhancement techniques (PETs).

Lithographic tool providers such as ASML (Veldhoven, The Netherlands) have developed their tools to enable Moore’s Law to continue. 

**Figure 13:**
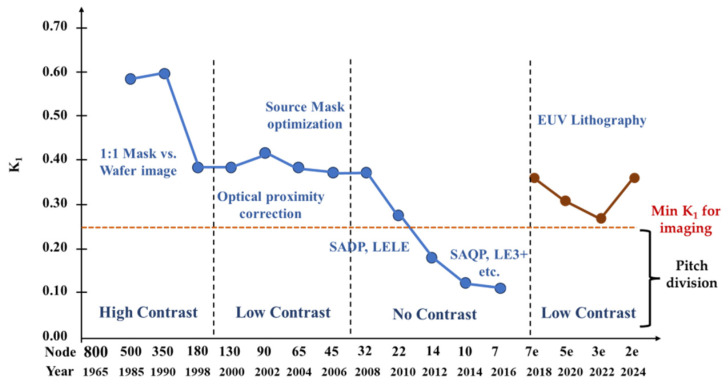
K1 factor evolution through the years of technological advancement.

The theoretical limit of the k1 is about 0.25. When k1 is smaller than 0.25, the image contrast is very low so that there is no image that can be printed. As Figure 13 shows, in earlier years, the k1 factor is above 0.5 when the imaging has high contrast, and there is no need to implement various PETs. When the k1 factor is below 0.5, it moves to the low-contrast era, where various PETs are required, such as optical proximity correction (OPC), and source mask optimization (SMO) [146,147,148]. In both high-contrast and low-contrast eras, the patterning has been achieved through single exposure and single etch approach. The wavelength has been reduced from i-line to KrF to ArF, and the NA has been enlarged to achieve the required resolution following Moore’s Law. 

Until sub-20 nm nodes are reached, the FinFET structure in the Logic/MPU process has been introduced [149]. At this technology node, the k1 factor is close to its theoretical limit of 0.25, using DUV ArFi lithographic tools. This is called the ‘No Contrast’ region, as Figure 13 shows. This means the traditional single litho-etch approach can no longer be used to shrink patterns [146,150]. EUV was not mature enough either by that time to continue the traditional approach. Double patterning and quadruple patterning thus have been developed as practical solutions to continue the node shrinkage. There are two major types of these multiple patterning techniques, litho-etch-litho-etch (LELE) and self-aligned double patterning (SADP) or self-aligned quadruple patterning (SAQP) [151]. In SADP or SAQP (SAmP), there are several steps of thin-film depositions and etchings to reduce the pitch. LELE is implemented for 2D structures such as contact holes via logic metal layers. SAmP is implemented for line and space patterns, such as Fin gate layers. After the SAmP processing steps, the lines are cut, or spaces are blocked, to form device patterns. 

### 4.1. EUV in Mass Production

EUV lithography has been used in chip mass production since 2019, when TSMC announced at the IEDM that EUV technology satisfied high volume production requirements; thus, they started the volume ramp of the enhanced 7 nm technology with EUV insertion [152]. In the same year, the first few consumer chips at 7 nm nodes with reference to EUV processing lithography, HUAWEI Kirin 990 Series 5G SoC (Shenzhen, China) [8,153], and Samsung Exynos 9825 (Suwon, Republic of Korea) [152], were also announced. EUV has been used in mass production at DRAM since Samsung’s announcement of the 1z process node [154]. Since the introduction of EUV, many major technical challenges have been overcome, i.e., source power, pellicle, tool availability, resist resolution, and mask manufacturing [155]. The EUV light source, EUV photoresistance, line width roughness (LWR), etc., have been introduced as nanomaterials references therein [155]. EUV is now part of the baseline manufacturing processes, as Wallace reported at 2020 SPIE [150]. To a large part, this is thanks to the excellent progress made for all areas of EUV lithography over the past several years. 

Since the successful EUV adoption in mass production [152,153,156], this has brought the k1 factor back to the low-contrast (k1 > 0.25) region from the no-contrast (k1 < 0.25) region, as shown in Figure 13. From 22 nm to 7 nm, there is no contrast and no imaging using single lithographic exposure, thus LELE and SAmP are used for pitch division. At the 7e (nm) node, the EUV is implemented, which brings the k1 factor back to the lower k1 > 0.25 region, as shown in Figure 13. In this lower k1 region, single EUV exposure (NA 0.33) is possible using various patterning enhancement techniques. At the 3e (nm) node, the EUV single exposure (NA 0.33) reaches a k1 factor of 0.25; thus, a high NA EUV (0.55) is needed to maintain a lower k1 single-exposure approach to form images.

EUV lithography can reduce the manufacturing complexity since it enables the single-exposure single-etch approach to pattern-critical layers. For the less critical layers, DUV has still been used to achieve overall cost effectiveness. Consequently, the lithography solutions need to support the mix and match use of EUV and DUV.

### 4.2. Overlay and Edge Placement Error (EPE) Challenges

With the continuous shrinkage of the design rules, the overlay requirement is becoming more and more tight. The mass production for advanced nodes requires overlay performance to be 2 nm and below in EUV platforms [157]. This is challenging for both overlay correction by scanner as well as overlay measurements. 

For the global overlay measurement, the IBO (image-based overlay) strategy has been widely used in mature products. As the technology node continues to shrink, the DBO (diffraction-based overlay) strategy has showed impressive performance. Studies suggest that IBO is better for non-tool-induced shift, while DBO is better for tool-induced shift [158,159]. However, IBO has faced more contrasting challenges when SADP and SAQP patterning film stacks have been introduced, while DBO has shown less film stack dependence with good overlay precision [160]. 

To support smaller technology nodes, DBO metrology marks have been reduced in size, called uDBOs. Thus, uDBOs have more flexibility to be placed not only in scribe lines, but also in primary higher-order intrafield corrections [158]. The uDBO metrology has also implemented muti-wavelength measurements. Compared with prior generations of dual-wavelength methodology, its accuracy and robustness to process variation have both been improved [159].

The second challenge in overlay is the cross-scanner platform matching between DUV and EUV. This is because the intrinsic design is different for sub-systems such as projection optics, reticle clamping, and wafer clamping for DUV and EUV systems. The current practice of EUV vs. DUV matching includes two steps with reasonable performance: I. Both EUV and DUV scanner grids need to be calibrated to a reference wafer grid which contains a dense intrafield layout. II. Fine-tuning is carried out for matching by using higher-order corrections per field on the EUV scanner [160]. 

**Figure 14:**
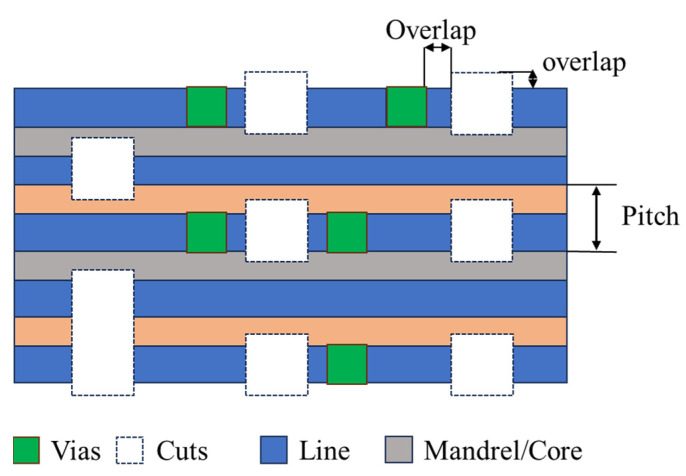
A schematic picture of an EPE as tolerance for the relative placement of two edges (litho cut, via, and spacer feature).

In the traditional single lithography and the single-etch approach, the critical parameters to control in the lithographic process are CD (critical dimension) and overlay variations. In a multi-patterning scheme, such as SAmP and LELE, a new parameter, EPE (edge placement error) is introduced. It combines CD, overlay errors, and includes local variations of CD and LWR (line width roughness) [160]. EPE is defined as the displacement of edges of two features from their target locations, as shown in Figure 14. In this example, the line/space grating (green color) is patterned using ArFi and a subsequent SAmP. The 2D geometry is created by cutting this grating pattern. The via layer is placed on top of line and next to the cut pattern. In lithography and SAmP, because of the processing variation, the displacement of the edges of the 2D cut/via can differ from the intended locations. This results in the overlap distance in both x and y direction are out of design distance, as shown in Figure 14. 

EPE can be calculated by an analytical method, as follows [160]:

$$3\sigma_{\mathrm{EPE}}=\sqrt{3\sigma_{overlay}^{2}+{\left(\frac{3\sigma_{CDU_{lines}}}{2}\right)}^{2}+{\left(\frac{3\sigma_{CDU_{cuts}}}{2}\right)}^{2}+\frac{3\sigma_{LWR_{lines}}^{2}+3\sigma_{LWR_{cuts}}^{2}}{2}}$$

This equation consists of overlay errors and global CD errors from the lines and the cuts. It also includes local CD errors such as LWR of lines and cuts. Apparently, in order to minimize error, EPE requires optimization across the scanner platform of the lithography processing steps. 

## 5. Epitaxy in Transistor Structures

### 5.1. Selective Epitaxy Growth (SEG) of SiGe in S/D Regions

**Figure 15:**
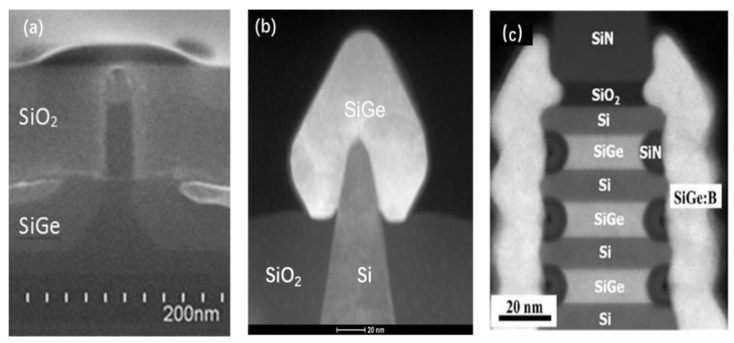
SEG SiGe in source and drain (S/D) regions in different structures: (**a**) 2D planar [170], (**b**) 3D FinFETs [7] and (**c**) GAAFETs [169]. Reprinted from ref. [7,169,170]. Copyright Elsevier 2016, AIP 2013, and IEEE 2022 Publisher.

As the technology node shrinks, strain engineering in the channel region is an effective technology to improve transistor performance. By depositing stressor material in S/D regions, strain is induced in the channel region which is one of the key factors in the development of aggressively scaled nano transistors. SiGe(B) and Si(P) materials are formed by SEG in the recessed S/D areas as the stressor materials in pMOS and nMOS, respectively. SEG of Si_0.83_Ge_0.17_ was initially integrated to enhance the carrier mobility in the 90 nm technology node [161]. To obtain more strain inside the channel, the Ge content of SiGe in S/D areas has been increased node by node to more than 50% [162,163]. This is a huge challenge, as the increase in Ge content is accompanied by an increase in epitaxial mismatch defects. At the same time, the S/D morphology was constantly adjusted as the device structure changes. The sigma (“∑”) shape of S/D was used to generate more strain in the channel area. This is because the SiGe films grew closer to the channel area [164,165,166]. Then, the morphology was changed from 2D planar to 3D FinFETs [167,168] and GAA Nanosheet FETs [169], as shown in Figure 15. 

The SEG of SiGe on Si Fins requires some issues to be paid attention to, namely, defect-induced strain relaxation [171], facet formation [172,173], and Si Fins damage. However, it is extremely difficult to obtain selectivity and induce the strain in the structure of GAA Nanosheet FETs, with the inner spacer SiN, HM (hard mask) SiO_2_ and SiN, and multi-Si nano sheets exposed. In the SEG process, the deposition of SiGe loses its selectivity on the SiN surface. Finally, the growth of SiGe film will cover the SiN inner spacer, and the topography will be discontinuous and unsmooth, which will affect the amount of strain generation [174].

**Figure 16:**
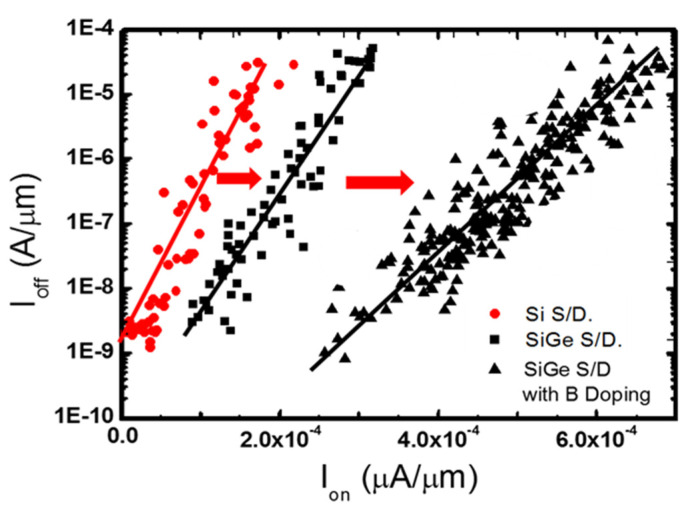
The I_ON_/I_OFF_ ratio of FinFET PMOS fabricated with different S/D devices [175]. Reprinted with permission from ref. [175]. Copyright Springer 2019 Publisher.

During the S/D SEG process, the B in situ doping was also applied in the S/D to reduce sheet resistance in the FinFET PMOS structure [175,176]. Figure 16 shows the I_ON_/I_OFF_ ratio comparison image of the FinFET PMOS devices. SiGe:B S/D devices show improved performance (I_ON_/I_OFF_ ratio, drive current), which is standard for the FinFET device. Although some strain in the SiGe film is compensated after B doping, Ion levels were increased when contact resistance declined in S/D. It also can be predicted that the decrease in SiGe:B contact resistance of S/D is the most key factor for the performance enhancement of the GAA NS transistors beyond the 5 nm node. One of the critical points for losing the strain is the formation of silicides. Ni is still the most suitable metal compared to other contact metals for the 5 nm technology node due to low Si consumption [177,178,179].

**Figure 17:**
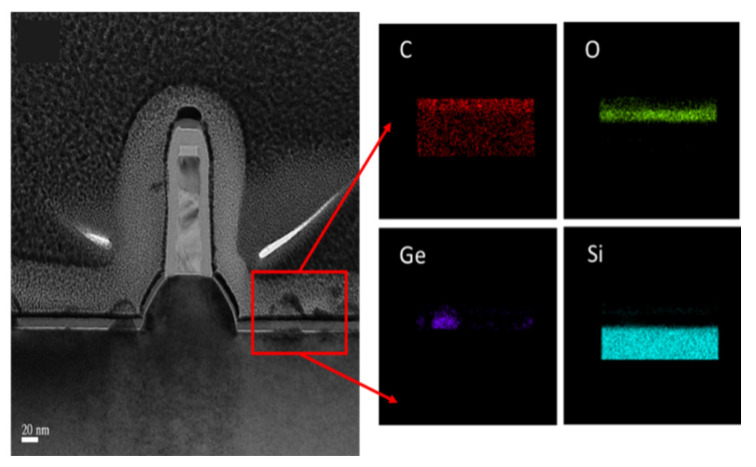
TEM cross-section image and EDS element mapping of relatively defected SiGe growth layer [171]. Reproduced from ref. [171], open access by Springer, 2017.

The quality of the initial Si surface has an important role in the quality of the SEG. This relates to the cleaning and thermal treatment of the exposed Si surface prior to the epitaxy. For the 2D planar and FinFETs structure, standard cleaning methods (SPM, APM, and DHF-last) are widely used to avoid any impurities or residues on the Si surface [171]. As shown in Figure 17, the C content of the Si surface is found between SiGe and Si, and SiGe film was grown independently [171,175]. These organic contaminations and polymers are not completely removed by standard cleaning, which leads to the poor growth of SiGe.

One of the important issues faced is contact resistance in S/D windows. This issue can be solved by forming silicides, but both the thermal budget and Si consumption are key roles in the transistor performance. Ni silicides have been widely used; moreover, the relaxation of the SiGe layer could be diminished by introducing carbon in the SiGe layers [176,177,178,179]. The SEG process of SiGe suffers from a pattern-dependency problem when, in the profile of the epi-layer, the exposed Si areas in openings (for example, in the S/D regions) in a chip differ for different layouts [19,20,21,38,180,181,182,183]. The main reason behind this problem is the non-uniform consumption of reactant gases (GeH_4_, SiH_4_, DCS, HCl) over a patterned wafer (global effect) or an array of openings inside a chip (local effect), when the layout of chip is changed. Until now, the pattern dependency of SEG in planar devices and FinFET devices has been systematically studied [180,181,182,183], but for GAA Nanosheet, devices have not been carried out due to the complexity of work. It is challenging to calculate and forecast the consumption of reactive gas in the 3D multiple stacks, not only because of size shrink and the density increase of the planar in the layout, but also because of the GAA device structure. A remedy to this problem is to introduce growth modeling which takes into account the chip layout and transistor’s structures in advance to predict the chip layout and growth parameters in a way that results in the uniform consumption of reactant gases during the growth period [184,185,186,187,188].

### 5.2. Epitaxy of GeSi and Ge for Channel Region

To improve the performance of MOSFETs, novel materials, new concepts, and new device architectures are under continuous research and development. Despite the rapid development of Si CMOS technology, there are several technical challenges currently faced, mainly including the gate controllability of electrical charges. To improve the gate control ability, FinFETs have been developed for the technology nodes beyond 22 nm. For the ultra-scaled sub-5 nm technology nodes, GAA nanowire (NW) transistors provide excellent gate control and immunity against the SCEs. After reviewing the evolution of Si transistors, ranging from planar MOSFETs to FinFETs and GAA NW FETs, device performance had been greatly improved via introducing group IV semiconductor materials, including SiGe, Ge, GeSn, etc. Other strategies include band engineering, heterostructure, and strain engineering, which are helpful for enhancing the carrier mobility via changing the carrier effective mass. Compared with traditional Si material, Ge (Sn) features better hole mobility due to the reduced effective mass. Thus, tremendous effort has been made to explore Ge (Sn)-related GAA NW FETs towards the sub-5 nm technology node. Up to now, there are only three groups that have demonstrated the presence of vertically stacked Ge (Sn) GAA NW FETs owing to the cutting-edge Ge (Sn) CVD growth technology (Table 3).

**Figure 18:**
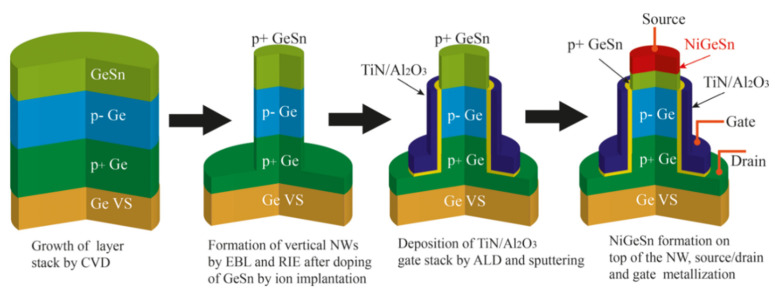
Schematic diagram of the GeSn/Ge heterostructure growth and vertically stacked GAA NW FETs process [192]. Reprinted with permission from ref. [192]. Copyright 2021 ACS Publisher.

After carefully considering the band alignment and strain engineering, M. Liu et al. [192] designed the GeSn/Ge heterostructure-based high-performance vertically stacked GAA NW FETs with traditional Si CMOS processing technology. The main growth processes are as follows: (I) growth of the intrinsic Ge layer on Si (100) substrate in an RPCVD reactor; (II) 200 nm thick p-type doped Ge layer growth (boron doping concentration: 2.5 × 10^19^ cm^−3^); (III) 150 nm thick slightly p-type doped Ge layer growth, which is used for channel length definition; (IV) 60 nm thick GeSn layer with 8% Sn incorporation. To realize the vertically stacked Ge_0.92_Sn_0.08_/Ge GAA NW FETs, the processes are schematically outlined in Figure 18. It should be noted that fabrication processes should be implemented at the low thermal budget, thus maintaining the structural stability for GeSn.

**Figure 19:**
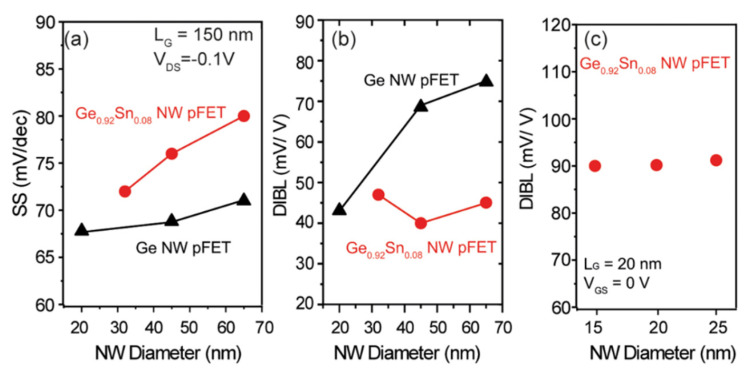
(**a**) Measured SS, (**b**) measured DIBL, and (**c**) simulated DIBL as a function of NW diameters [192]. Reprinted with permission from ref. [192]. Copyright 2021 ACS Publisher.

To study the diameter effect on the performance of Ge_0.92_Sn_0.8_/Ge GAA NW FETs, NWs with three diameters were fabricated. Electrical characteristics, such as SS and DIBL on the NWs, were extensively characterized. Compared with the Ge NW devices, GeSn/Ge NW devices with a gate length of 150 nm exhibit higher SS due to the lower thermal budget limitation of the GeSn/Ge heterostructure and higher density of interface traps (D_it_) (Figure 19a). With the reduced NW diameter, GeSn/Ge NW devices feature steeper SS than that of their Ge counterpart, which can be traced back to the improved gate control ability. Moreover, the DIBL behavior of GeSn/Ge NW devices was further compared to the Ge NW pFETs. It is highly expected that Cit should be higher than Ge NW pFETs, owing to the lower thermal budget, which, therefore, displays a lower DIBL (Figure 19b). Therefore, the GeSn/Ge heterostructure yields a better performance compared to the Ge homojunction. To verify the experimental DIBL results, DIBL values were also extracted from the simulation (Figure 19c). As a result, the simulated DIBL values are 40–50 mV/V, which is larger than the experimental data. Nevertheless, the qualitative performance is well reproduceable [194].

### 5.3. Growth of SiGe/Si for Gate-All-Around (GAA) Structures

In both vertical and horizontal GAA structures, fabricating nanowire or nanosheet channels from Si/SiGe multilayers is widely used because of advantages in the process and material properties. Si and SiGe are compatible with traditional Si-based processes. High-quality Si/SiGe stacks can be epitaxially grown on Si substrates. The Si/SiGe stack has high flexibility in its process. By adjusting the growth sequence and Ge content of the Si/SiGe stack, different channel materials can be obtained, such as Si, Si_0.7_Ge_0.3_, and Si_0.2_Ge_0.8_ [195,196]. Apart from that, the high mobility of Ge can boost the electrical performance of the device.

In terms of process, both Si and SiGe can be used as the channel, which depends on the structure of the Si/Ge thin film material and the etching process [197,198,199]. It is worth mentioning that if you want to use SiGe as the sacrificial layer and Si as the channel, then epitaxial growth of low Ge components is recommended, as this avoids creating defects during the growth process. Another advantage is the flexibility of the nanosheet width.

**Figure 20:**
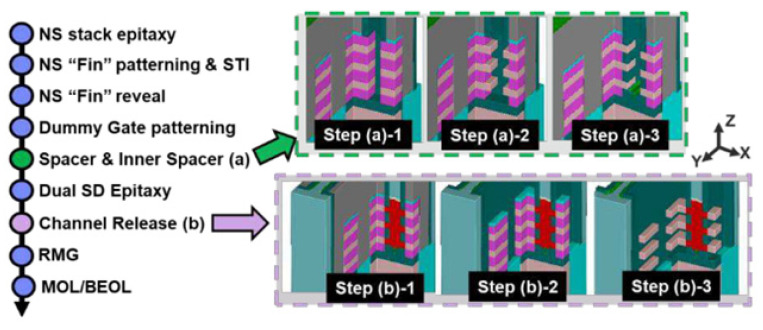
A schematic of a multilayer nanosheet GAA process sequence. (**a**) IS formation, and (**b**) CR process steps illustrated along dummy gate in x-direction, and along W_NS_/“Fin” in y-direction [200]. Reprinted with permission from ref. [200]. Copyright 2021 IOP Publisher.

The deposition of Si/SiGe MLs and the subsequent etching process are the main differences between conventional FinFET and GAAFET fabrication, as shown in Figure 20 [200]. The process of Si/SiGe epitaxial growth is an important part in advanced CMOS fabrication.

The commonly used growth methods of the Si/SiGe stack are reduced pressure chemical vapor deposition (RPCVD) [2] and molecular beam epitaxy (MBE) [201]. Considering the subsequent process, what is required is Si/SiGe stacks with sharp interfaces and high quality. The thermal budget also should be considered [202,203].

#### Different Novel Epitaxial Si/SiGe Is Used as Channel to Boost the Device Performance

Starting from the epitaxy of Si_0.7_Ge_0.3_/Si multilayers, S. Barraud et al. reported a two-stacked Si nanosheet [204]. The thickness of the Si and SiGe layer was chosen at 9 nm to ensure the good crystallinity of epi-layers. SiGe was used as the sacrificed layer to form the Si channel. Similarly, a seven-level-stacked Si nanosheet GAA transistor was fabricated. Extremely high current drivability (I_sat_ = 3 mA/µm at V_DD_ = 1 V), and electrostatic control (SS_sat_ = 64 mV/dec) have been shown in such devices [205].

A pTFET with a Si_0.2_Ge_0.8_ nanosheet of 120 nm in width containing SiGe/Si multilayers as active regions (SiGe is the channel material) have been manufactured [206]. Si sacrificial layers are removed using wet etching, and a Ge condensation process was conducted to obtain Si_0.2_Ge_0.8_ NS. The device shows 50 mV/dec minimum SS and 70 mV/dec average SS. The I_ON_ is 69.2 nA/μm (@V_DS_ = 0.7 V), and I_OFF_is 800 fA/μm (@V_DS_ = −0.7 V).

To decrease the sub-channel leakage and to obtain an improvement for power performance as well as minor process variation in GAA structure, J. Zhang et al. used a strategy called full-bottom dielectric isolation (BDI) [207]. This is achieved through a novel Si/SiGe stack structure. They grew an SiGe layer with high Ge% content as the initial layer on the substrate, and then SiGe/Si stacked layers with low Ge% content were grown. The SiGe layer with Ge content was used as a sacrificial layer.

**Figure 21:**
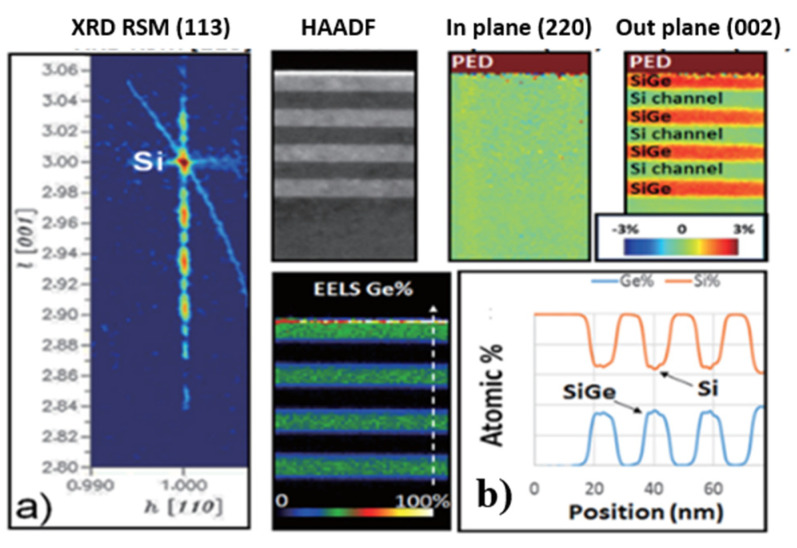
(**a**) X-ray diffraction reciprocal space mapping (XRD-RSM), (**b**) precession electron diffraction, and electron energy loss spectroscopy (EELS) of the SiGe/Si stack [174]. Reprinted with permission from ref. [174]. Copyright 2017 IEEE Publisher.

**Figure 22:**
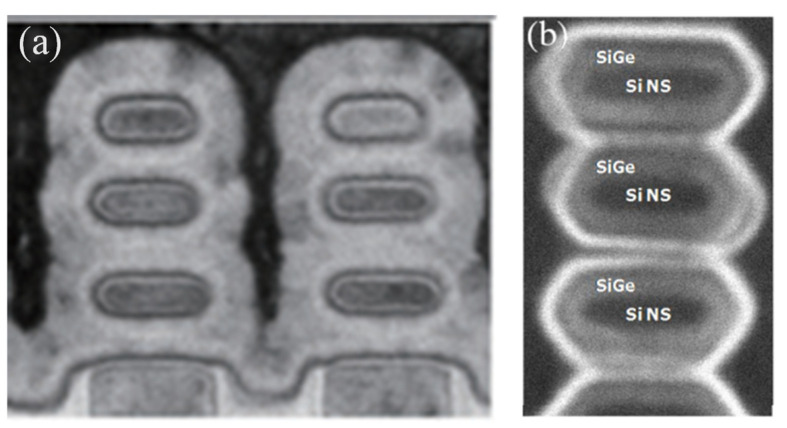
(**a**) TEM images of Si NS after replacement metal gate (RMG) [174], and (**b**) TEM images along the gate of the SiGe cladding layer [210]. Reprinted with permission from ref. [174,210]. Copyright 2017, 2020 IEEE Publisher.

Loubet et al. investigated SiGe/Si superlattice stacks to process Si channels. Superlattice SiGe/Si multilayers were epitaxially grown to form a nanosheet [174]. As shown in Figure 21a,b, the multilayers are pseudomorphic, with compressively strained SiGe layers and unstrained Si layers. The NS structure is shown in Figure 22a.

On the basis of this research, later, J. Zhang et al. also investigated mechanical behavior in fin-patterned SiGe/Si multilayer structures [207]. Strain maps obtained by GPA show that the strain in the SiGe layer is released after fin-patterning, causing compressive strain in Si. S. Rabah et al. further studied the evolution of strains over the integration of Si-NS GAA [208]. It is shown that Si goes from unstrained to slightly tensile after fin-patterning, which is also consistent with ref. [209]. Furthermore, the recess of S/D results in the strain in Si and, later, the tensile stress is released after releasing the channels. A transition from compressive to tensile strain in SiGe/Si NS is demonstrated. SiGe/Si multilayers with Ge contents of 60% and 35% were studied. The results showed that the final stress amount is related to the Ge content of SiGe/Si multilayers.

S. Mochizuki et al. made some changes after releasing the channel and using a compressive SiGe channel for pFET [210]. Two major steps are different, with normal Si NS flow after epitaxial of SiGe/Si: Si NS trimming and SiGe selective epitaxial growth. As a result of Si nanosheet trimming and SiGe epitaxial growth on the trimmed Si nanosheet, an SiGe cladded Si nanosheet was fabricated. The structure is shown in Figure 22b. The SiGe cladded nanosheet increased hole mobility and reduced V_T_. Additionally, this group later achieved a highly compressive strain in the SiGe cladded nanosheet channel by adjusting the thickness of the cladded SiGe layer and the Ge content [211]. A further improvement in hole mobility and channel resistance reduction were observed.

## 6. Implantation and Advanced Doping Methods

Ever since the structure of CMOS transistors evolved from planar to 3D, e.g., inFinFETs, and even to GAA devices, homogenic doping on each side of the structure has become a challenge for the doping process [212]. Large-angle tilt implantation had been employed to realize a relatively uniform dopant distribution on both sides of the wall and the top area of a fin structure. However, for the 5 nm node and beyond, to ensure better gate control, gate all around devices like nano-sheet will take the place of FinFET gradually. Thus, the doping on the bottom area must be considered as well. Additionally, for that reason, some novel doping methods such as plasma doping and solid-state diffusion were developed. Moreover, the application of implantation was also extended by co-implantation such as in the case of Gallium to improve the contact resistance of the device.

### 6.1. Plasma Doping

Plasma doping is designed for high-dose ultra-low energy or high-aspect-ratio structure doping. It has been proven to be effective in forming ultra-shallow junctions; the whole wafer was immersed in dopant-contained plasma to obtain a conformal doping profile [212,213,214,215]. However, as there was a bias in the PLAD system to accelerate the ions, the maximum doping concentration still appears on the top area of the device and leads to nonuniform distribution. In addition, the collision between the accelerated ions and the substrate might cause lattice damage to some extent.

Recently, another plasma-related doping process named plasma-assisted doping (PaD) has been conducted to overcome the limitation of conventional implantation processes [216]. In order to reduce the surface damage created by the accelerated ion, the bias voltage in standard PLAD systems was removed. In this condition, both the damage to the substrate and doping uniformity could be improved. In the PaD process, the dopants transmit into the substrate lattice via the vacancies during doping process. There are about three steps in a PaD process. At the beginning, H plasma is generated by a low power to treat the surface of sample to obtain a high vacancy concentration near the surface area; in the following process, dopant precursor gas is ionized where the vacancies are diffused into the substrate. In the end, in situ capping layer (SiN) deposition is necessary to prevent dopant diffusion and contamination in the annealing procedure.

### 6.2. Solid-State Diffusion

Another potential solution for conformal doping is by introducing the impurities into the substrates from solid sources such as PSG or BSG films. In this method, an ultra-thin dopant-incorporated film could be deposited by ALD or CVD, etc. [217,218]. It is well known that the incident angle of implantation is restricted by the shadowing effect due to the increase in packing density. Therefore, thin film deposition has better step coverage on the whole surface of the 3D structure. Dopant-contained glasses could be a solution for conformal doping; when the deposition was completed, the dopant was then pushed into the substrate and activated under a drive-in annealing. To prevent the dopant from out diffusion, a capping layer is necessary to be deposited before the annealing. The experimental results show that the thickness of both the capping layer and the doping layer has a great impact on the final doping level of substrate [217]. For example, the concentration of Phosphorus could be improved by increasing the thickness of the PSG film, and thicker the capping layer, less dopant diffused out. As a result, the surface dopant concentration is close to 10^20^/cm^−3^, which just satisfies the doping requirement for ultra-shallow junctions. Moreover, there is no ion bombardment in the procedure, then there is nearly no damage in the doped area.

Another doping method is called mono-layer doping (MLD). In this method, the dopant molecules are attached uniformly on the surface through a series of chemical treatment steps, and the doping level could be well-controlled by tuning the dopant composition of the molecules.

Except for the requirement of conformality, parasitic resistance is another challenge that needs to be addressed. As the critical dimension of devices decreases, the contact area decreases accordingly, and it is necessary to achieve a higher dopant activation level in S/D to lower the contact resistivity [214,219,220,221,222,223]. At present, the S/D of PMOS are p-type doped SiGe epitaxial films where the doping procedure is accomplished by boron in situ doping or implantation. However, p-type doping in SiGe has been a challenge for its application beyond 5 nm. An alternative solution for this is the incorporation of Gallium together with Boron, which are then activated by nanosecond laser annealing, which is believed to be one of the most promising annealing methods. As an element of ground III, both B and Ga were p-type dopant species for Ge. B shows higher active doping concentrations than Ga when they are activated by a traditional rapid thermal treatment such as RTP. However, a combination of Ga and B implantation with nanosecond laser annealing shows a lower contact resistivity. Under a determined annealing condition, the surface of the SiGe could be transmitted to melted status by nanosecond irradiation of the UV laser, and the Si concentration of this area will be increased and lead to segregation of Ga and Ge towards the melted region.

In conclusion, the doping process is still a challenge for the development of CMOS devices at 5 nm and beyond. Therefore, more conformity, less damage, and a high activation level will be the key points to be investigated in the future.

## 7. HKMG, ALD Technique and Negative Capacitance Materials

High-k dielectric and metal gate material are introduced into CMOS device by intel at the 45 nm technology node in order to improve the device performance. A series of characteristics are required for the successful implementation of high-k dielectrics, such as a high ε-value, a large band gap and band offsets with Si, low interfacial trap density, thermodynamic stability with Si, etc., [224]. Metal gates should have suitable work function in order to realize the required VT. W. Xiong et al. reported the work function targets for different devices [225]. The work function for planar bulk high-performance NMOS and PMOS should be 4.2 eV and 5.0 eV, respectively. For multi-gate devices, due to the better gate control ability, the work function is about 4.4 eV and 4.85 eV for high-performance NMOS and PMOS, respectively.

Thin SiO_2_ is used as interfacial layer and HfO_2_ is chosen as a dielectric for industrial production. TiN is used as a PMOS work function metal, and TiAl-based alloys are used as NMOS work function metals. HfO_2_ is grown by ALD (atomic layer deposition). The interfacial layer is usually a chemical oxide grown by wet cleaning, which can provide a –OH-terminated surface. The –OH provides an active chemical group for the adsorption of the ALD precursor. This can lead to good linear growth of HfO_2_ without incubation. The traditional growth method for the metal gate is CVD or PVD. However, when the device structure evolves from planar structure to 3D FinFET and GAA architecture, CVD or PVD can no longer realize conformal step coverage. Therefore, ALD gives the best solution due to its excellent filling capability at even nanometer-range thickness. TiN, TiAlC, and TiAl have been successfully grown by ALD, which can meet the V_T_ requirement [226,227,228,229,230,231]. Along with the shrinking of the feature size, the material system of high-k dielectrics and metal gates is nearly unchanged. The relative thickness tends to be decreased [170,232].

The device dimension continues to scale down, but the high-k and the metal gate layers cannot always follow this rule. Thus, the volume-free approach for multi-V_T_ is necessary. It is known that the effective work function of the metal has dependence on its thickness. It is reported that a range of 150 to 200 mV V_T_ adjustment is achievable through thickness modulation in high-k and metal gate stacks [233]. Conformality and thickness control are very critical. Another V_T_ tuning method is by electrostatic dipole, which needs relatively less volume and can give more flexibility to the integration. The interaction of the oxide dipole layer with its interfacial layer creates a V_T_ shift, resulting in their group electronegativity difference [233,234]. The dipole multi-V_T_ modulation was conducted by R. Bao et al. with ALD La_2_O_3_, followed by drive-in annealing before metal gate deposition in the FinFET device [235]. There is a shift of more than 200 mV V_TS_ from the reference V_TS_ device by using different La_2_O_3_ thicknesses. This dipole multi-V_T_ can also be combined with metal multi-V_T_ to have more flexibility to offer more VTs. S. Hung et al. placed a thin LaOx layer between the high-k dielectric and TiN, and then carried out a thermal drive-in process to form dipole at the interface of high k and the interfacial layer. About 0.8 nm LaO_x_ can provide ~300 mV of V_T_ adjustment [228]. K.L. Lee et al. noted 210 ± 25 mV as a V_fb_ improvement at only 0.4–0.5 nm physical and 0.1 nm electrical thickness, employing a new AlN cap on HfSiO [236]. This makes the integration more scalable. The AlN cap characteristics are nearly independent of growth method (ALD or plasma-enhanced ALD) if good nucleation of the initial layer is ensured. The technology of the interfacial Al_2_O_3_ doping HfO_2_ laminated stacked layer via controlled ALD cycles was systematically investigated by R. Xu et al. [237]. As the amount of interfacial Al_2_O_3_ doping increases, V_fb_ gradually increases to saturation. A shift of 270 mV V_fb_ was realized at 0.525 nm Al_2_O_3_ thickness.

## 8. Advanced Etching for Nano-Transistor Structures

### 8.1. Dry Etching for 3D Transistor

To suppress the SCEs caused by device miniaturization, from the 14 nm technology node to sub-5 nm, the transistor architecture has been experienced from FinFET to form GAA nanowires and nanosheets [238].

FinFET will always be the main structural form of the core device in many nodes below 14 nm. Recently, many special etching technologies have emerged to continue the vitality of FinFET technology [239,240]. For example, a special dry etching scheme is used to obtain ultra-high and steep fin in order to improve the performance of the device [241]. There are also methods used to trim fin by isotropic selective etching [242]. There is also special-shaped fin etching, which uses oxidation to obtain isolation from the substrate to achieve the effect of SOI [243], and scallop to obtain better gate control [244,245]. In addition, facing the etching countermeasures of new channel materials, such as Ge and SiGe and SiGe/Ge FinFET [246,247], hydrogen plasma is introduced as a new way to control morphology and CD (critical dimension) [248].

For core devices below 3 nm, nanowires or nanosheets in the form of GAA (the Si/SiGe superlattice structure) are considered to be the best device form to replace FinFET. GAA preparation technology based on FinFET architecture is considered as the most promising solution [249]. However, channel release and inner spacers are challenging processes not found in conventional FinFET [239].

**Figure 23:**
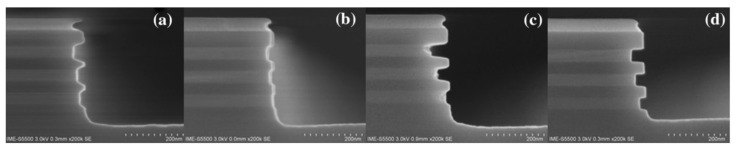
SEM cross-section micrographs of SiGe/Si multilayers after isotropic etching with reactant gas CF_4_/O_2_/He flow ratios of: (**a**) 1:0:0, (**b**) 1:1:0, (**c**) 4:1:0, and (**d**) 4:1:5 [253]. Reprinted with permission from ref. [253]. Copyright 2020 Springer Publisher.

For channel release, high selectivity and isotropy are the process requirements. Wet etching is not suitable mainly due to the capillary effect, which easily leads to structural collapse [250]. Vapor etching using HCl has also the limitation of crystal orientation selectivity [251]. Therefore, dry etching is a suitable technology for overcoming the abovementioned limitations. Most of the research reports propose remote plasma etching as a promising etching method [252]. Based on the traditional inductively coupled plasma etching machine, better etching results can also be obtained by using the optimized process as shown in Figure 23a–d [253].

**Figure 24:**
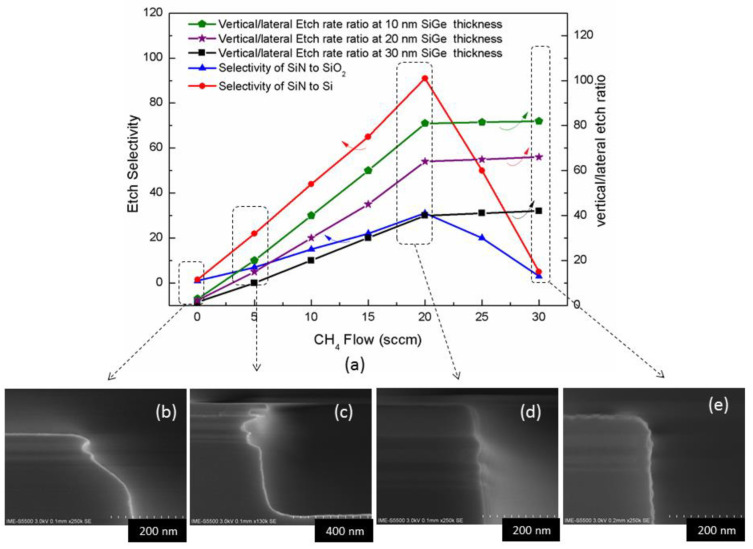
Influence on etching profile due to CH_4_ flow: (**a**) the dependence of vertical/lateral etch ratio and etch selectivity on CH_4_ flow; (**b**) etching profile with no CH_4_ flow; and with CH_4_ flow of (**c**) 5 sccm; (**d**) 20 sccm; (**e**) and 30 sccm [255]. Reproduced from [255], open access by MDPI, 2020.

The inner spacer is a process module used to balance parasitic capacitance and parasitic resistance in GAA process [254]. Challenges related to its etching include accurate control of cavity etching and anisotropic dielectric materials with high selectivity. A good etching effect can be obtained by selecting the optimized CH_2_F_2_/CH_4_/O_2_/Ar system as shown in Figure 24a–d [255].

### 8.2. Precise Etching: Atomic Layer Etching (ALE)

For horizontally stacked GAA nanowires or nanosheets, the inner spacer needs to accurately control the thickness to control the effective gate length; thus, it is necessary to accurately etch isotropic SiGe and anisotropic dielectric materials [255]. For vertical channel GAA (V-GAA), isotropic etching SiGe, controlling the channel diameter, and anisotropic accurate etching of the metal gate and high-k material are very important. Similar to the atomic layer deposition technology, atomic layer etching technology is a processing technology of great significance to advanced processes [256].

**Figure 25:**
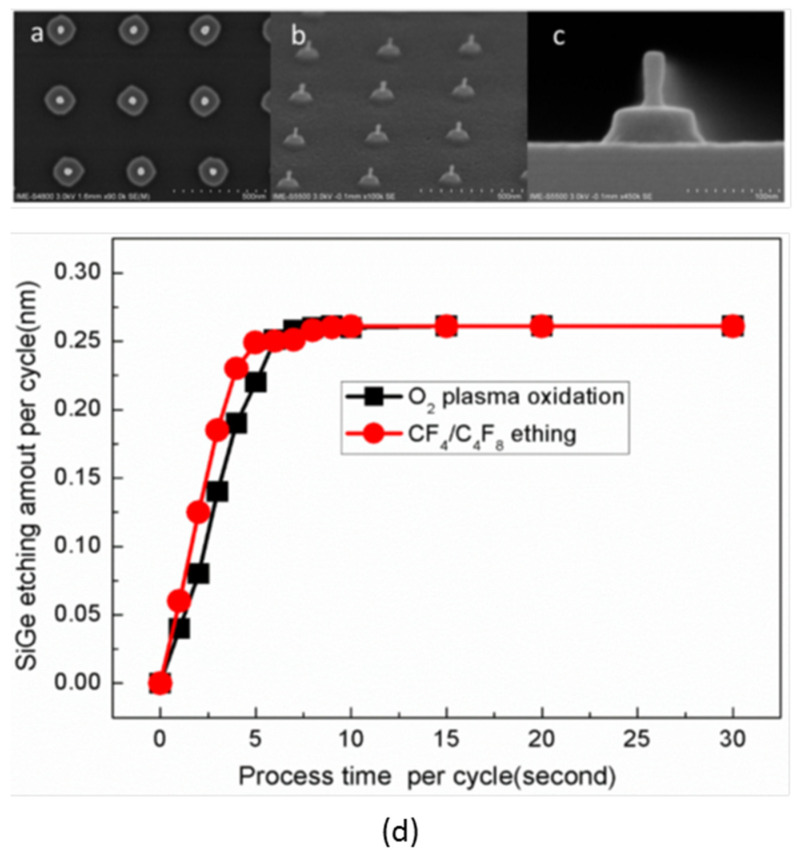
SEM micrographs of nanowires with diameter smaller than 20 nm after 100 cycles ALE using SiO_2_ hard mask: (**a**) bird’s-eye top view; (**b**) 45° tilt top view; (**c**) cross-section image; (**d**) Si_0.72_Ge_0.28_ etching for different process time per cycle [260]. Reproduced from [260], open access by MDPI, 2020.

The ALE wet etching of SiGe mainly includes a combination of self-limiting oxidation and oxide layer removal, mainly using a combination of H_2_O_2_ or HNO_3_ oxidation and HF/buffered oxide etching (BOE) [257,258]. Because wet etching has the limitations described in the previous section, the focus is on dry etching. The solution of O_2_ plasma oxidation and NF_3_/NH_3_/O_2_ oxide layer removal achieves the effect of digital high selectivity etching, but because there is no self-limiting feature, the etching accuracy of each cycle is not high [259]. Because the scheme using O_2_ plasma oxidation and CF_4_/C_4_F_8_ oxide layer removal is self-limiting, the etching accuracy reaches about 0.3 nm/cycle, as shown in Figure 25 [260]. Furthermore, the thermal ALE of SiGe has been shown to be dependent on the sequential exposure of oxygen (O_2_) or ozone (O_3_), hydrogen fluoride (HF), and trimethyl aluminum [Al(CH_3_)_3_], with an accuracy of 0.057 nm per cycle [261].

For ALE etching of SiN-based dielectric materials, the combination of downstream CHF_3_/O_2_ plasma modification and >100 °C heat treatment can achieve an etching accuracy of 2.8 nm/cycle [262]. For ALE using thermal reactions, the accuracy can reach 0.02 nm/cycle [263]. In addition, atomic layer etching of 0.9 nm/cycle can be achieved by the combination of CH_2_F_2_/O_2_/Ar modification and infrared heat treatment [264]. There are many reports about how to obtain selectivity between ALE SiN and silicon oxide [265]. ALE etching of high-K [266] and metal materials [267,268] has gradually become a research hotspot to better support advanced processes such as V-GAA [269,270,271].

## 9. Wet Etch and Cleaning

Due to their improved mobilities, SiGe, Ge, and III–V materials have mobilities of—40,000 cm^2^·V^−1^s^−1^ for InGaAs (electron) and 1900 cm^2^·V^−1^s^−1^ for Ge [1] (hole) compared to 1400 cm^2^·V^−1^s^−1^ for electrons and 450 cm^2^·V^−1^s^−1^ for hole of Si [272]. By entering the 10 nm technology node, the pure Si channel has been replaced with the abovementioned materials as listed. The device shape changes from fin-like to lateral GAA (LGAA) and vertical GAA (VGAA) for better channel control for the 5 nm node and beyond. It has been also reported that the nanosheet devices have better power performance than finfet 273. Meanwhile, the device fabrication of the 5 nm node and beyond considers not only the selective etching of sacrificial materials to the channel ones, but also prevents the collapse of the closely stacked channel material’s pattern during the nanowire/nanosheet release processes 273.

To overcome the high-temperature annealing processes 274, poly crystalline Si is used as the dummy gate and silicon dioxide as the dummy gate oxide material to occupy the space of the real gate and gate oxide. After the high-temperature annealing processes, the dummy gate of poly crystalline Si was etched away by metal alkaline solutions. Since the RMG is used to avoid crystallizations of the high-k dielectric during the rapid thermal annealing (RTA) process for dopant activation, this is successfully prevented in order to increase the leakage current of the gates 274. On the other hand, RMG prevents the chemical reactions between the metal gate and the high-k in RTA processes 275; furthermore, it avoids the boron diffusion into high-k 276.

**Figure 26:**
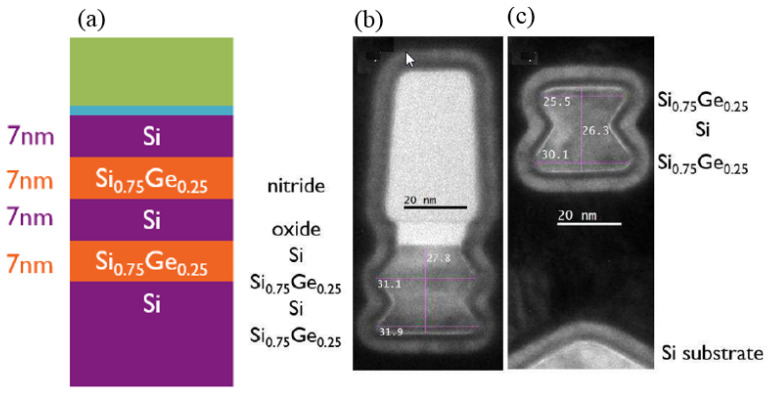
(**a**) Schematic illustration of a multilayer of Si_0.75_Ge_0.25_/Si nanowires applied for selective etching and TEM across image of 30 nm wide Si-Si_0.75_Ge_0.25_ nanowires after selective etching of Si (**b**) in TMAH 5% and (**c**) without the oxide–nitride hard mask 284. Reprinted with permission from ref. [279]. Copyright 2015, IOP Publisher.

In the 7 nm and 5 nm technology node, the RMG process is still applied to selectively remove the sacrificial Si material from the SiGe channel 277. Increasing Ge content in SiGe could greatly decrease the alkaline etch rate of SiGe, which enables the selective removal of Si to Si_0.75_Ge_0.25_ [273,274,275,276]. Si etching behavior in the alkaline solution is also well understood [277,278]. It is well known that the etch rate of the Si (111) direction is much slower than Si (001) and (110) crystallographic planes 284. With a TMAH 5% solution at 60 °C, the material underneath Si of the NW stack can be selectively etched away without losing SiGe, as shown in Figure 26. The 7 nm thick Si layers, which are sandwiched in between the Si_0.75_Ge_0.25_NWs, are removed until the (111) limiting planes are formed.

**Figure 27:**
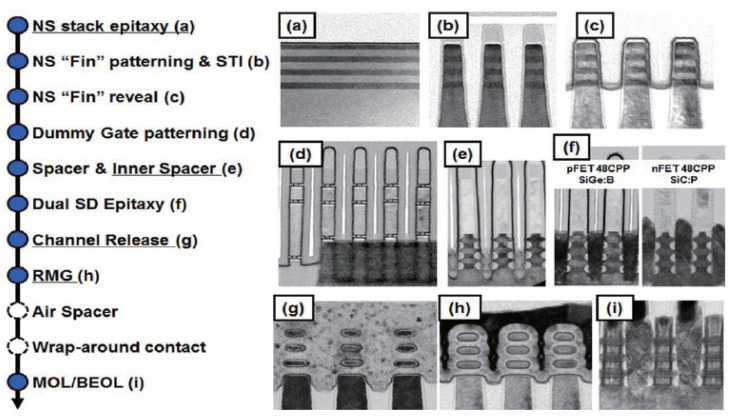
Multilayer of nanosheet channel is released by applying HCl vapor [282]. Reprinted with permission from ref. [282]. Copyright 2017, IEEE Publisher.

By using a conventional alkaline Si etchant such as TMAH (aq), it becomes difficult to remove the Si sacrificial layer from the SiGe stack during SiGe NW processing. The selectivity of Si and. SiGe etching is not high enough [280,281]. ACT^®^ SG-201, containing a surface modifier, could improve the relative etch rates of Si (110) and Si (111) orientations, and result in etching selectivity of Si (110)/Si (100) in the range of 1 to 2.5 and Si (111)/Si (100) of about 0.5 or above. The selectivity of Si (111)/SiGe 25% is significantly improved compared to the conventional Si etchants with the help of the Si surface modifier and an effective SiGe corrosion inhibitor in ACT^®^ SG-201. Consequently, ACT^®^ SG-201 is able to more efficiently to etch the sacrificial Si layer in the SiGe/Si multilayers 287. The reduced Si etch rate anisotropy in combination with an effective SiGe corrosion inhibitor avoids the loss of the SiGe layer during the nanowire release 287 (Figure 27).

For devices with the 5 nm technology node and beyond, both dry etching [282] and wet etching [174,283] have been investigated. Using the dry-etching method, the stacked nanosheet channel release was well released using vapor-phase HCl [282]. In the case of wet etching, we use aqueous solutions which have water as the solvent, and, finally, the wafers are rinsed with deionized water (DIW) or ultrapure water (UPW) to remove the residual chemicals from the substrate surface [283,284,285,286]. Due to the surface tension of water, different defects may appear. For instance, the high capillary force of water could pull nearby structures to form permanent imperfections during the drying process, which are known as pattern collapse 292, or stiction 293 in micro electro-mechanical systems (MEMS). To minimize the surface tension, liquid isopropanol (IPA) was used 295, but this turned out to be ineffective as the pattern spacing decreased and the aspect ratio increased.

**Figure 28:**
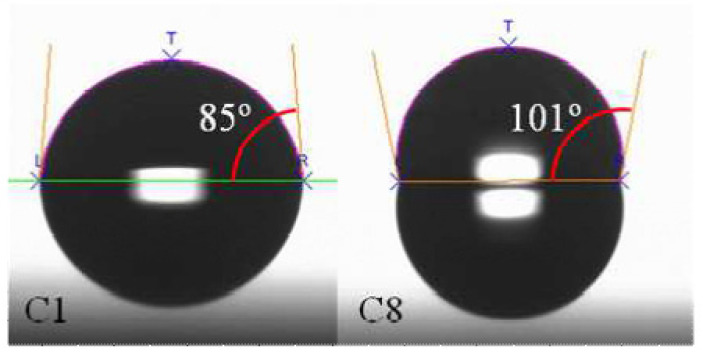
Illustration of contact angle test of C1 and C8 (the straight-chain alkyl group in the agents has carbon numbers of 1 and 8, respectively) 297. Reprinted with permission from ref. [287]. Copyright 2017, IOP Publisher.

The adhesive force (due to the capillary force) which pulls the neighboring high-aspect-ratio (HAR) structures overcomes the elastic force to generate stiction. By introducing lower-surface-tension liquids like isopropyl alcohol (IPA) to replace the water, the capillary forces can be effectively minimized during the drying process. The hydroxyl groups are considered as highly reactive, and are often seen from the silicon oxide surfaces after SC1 or ozonated water treatment in the field of microfabrication. Replacing the hydroxyl groups with inactive molecules like silylation agents, therefore, is a good solution to minimize the stiction 297. For drying HAR structure evaluation, two kinds of straight-chain alkyl group were compared. The straight-chain alkyl group in the agents possesses a carbon number of 1 and 8; therefore, they are noted as C1 and C8, respectively 297. The contact angle of C1 (85°) is measured as lower than that of C8 (101°), as illustrated in Figure 28. This is important to mention here as it is known that longer straight-chain alkyl groups result in the sample surface showing more hydrophobic behavior 297.

**Figure 29:**
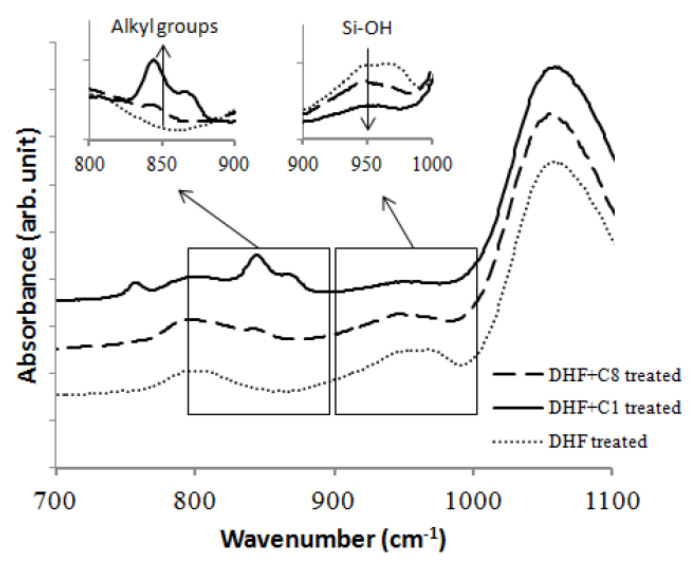
ATR-IR spectrum obtained from SiO_2_ powder after C1 or C8 treatment demonstrates that C1 is better due to higher hydrocarbon groups around 750 to 900 cm^−1^, whereas lower hydroxyl group is at 960 cm^−1^ 297. Reprinted with permission from ref. [287]. Copyright 2017, IOP Publisher.

**Figure 30:**
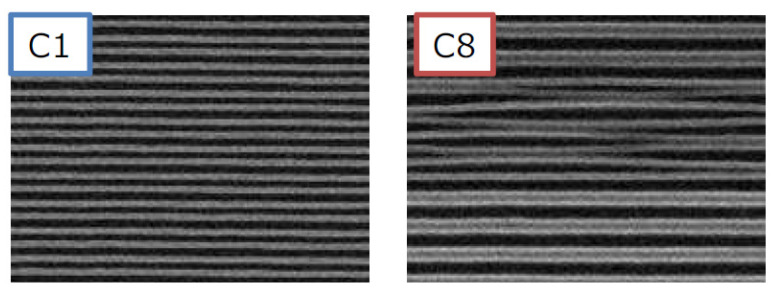
Top view micrographs of wafers after C1 and C8 treatment and drying 297. Reprinted with permission from ref. [287]. Copyright 2017, IOP Publisher.

However, the samples of silicon oxide power treated with C1 and C8 showed different results. As shown in Figure 29, that C1 treatment sufficiently eliminates the hydroxyl groups by measuring with attenuated total reflection–infrared spectroscopy (ATR-FTIR). In these experiments, the C8 reaction was suppressed by its steric hindrance; as a result, the existing hydroxyl groups could not be efficiently replaced with alkyl groups [287]. The drying process of the HAR structures with an aspect ratio of 13.3 applying C1 and C8 showed that it is not crucial to obtain high water repellency; however, it is significant to obtain lower surface free energy, as illustrated in Figure 30 [42]. The alkyl group surface can be simply removed by oxidative or reductive plasma strip by using N2O or N2/H2 reactant gases [288]. Farid et al. reported the formation of STI patterns with 9 nm CD, 25 nm pitch, and 160 nm fin height [288]. Therefore, it is expected that wet etching can be applied for the 5 nm node and beyond, as well as dry etching, to avoid any stiction of the nanostructures.

## 10. Metal Materials Interconnect

Copper has been replacing aluminum as a BEOL interconnecting metal material for over 20 years. However, copper interconnection faces many difficulties in balancing line resistance (line R) and reliability for advanced technology nodes [289]. Some strategies have been investigated to improve via and line resistance as well as reliability at small dimensions [290].

The self-forming barrier layer uses the seed layer of copper-based alloy to form a barrier layer through post-metalization annealing. The alloy elements in the experiment include V, Al, and Mn [291,292,293,294,295]. A widely considered promising copper interconnection method is based on the concept of a through-cobalt self-forming barrier (tCoSFB), in which Mn atoms in the doped copper seed layer diffuse through a thin cobalt liner and react with Si, O, and Ta to form a strong diffusion barrier layer at the interface between the liner and the dielectric. The concentration of Mn in the CuMn target material can vary between 0.5% and 10%; the final concentration value depends on a comprehensive evaluation of the line resistance and reliability [296]. Nogami et al. believed PVD/ALD TaN and/or tCoSFB as a viable solution to continue scaling barrier/wetting layers, and the interconnection resistance of metals such as cobalt and ruthenium will be better than that of copper due to the limitation of the reduced thickness of the barrier layer in the 5 nm technology node [289].

**Figure 31:**
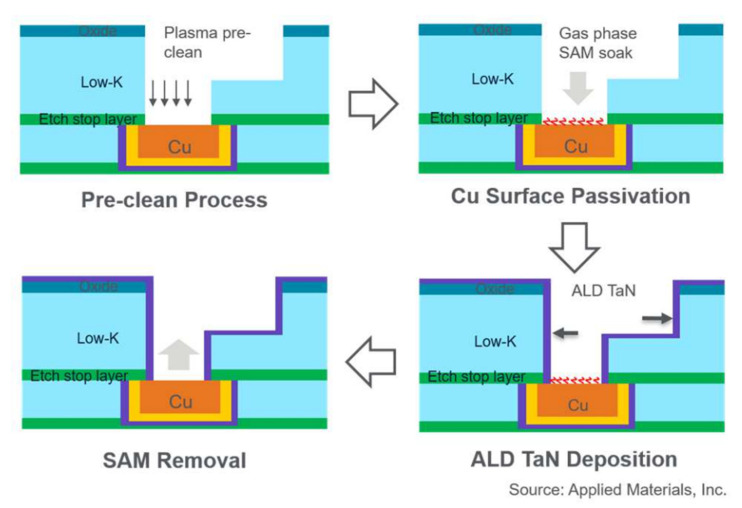
Schematic pictures showing the integration flow of selective barrier in Cu interconnection [297]. Reprinted with permission from ref. [297]. Copyright 2021, IEEE Publisher.

Gas-phase self-assembled monolayers (SAM) only provide highly selective adsorption on metal. Based on the reasons given above, a new integration method using a selective barrier is invented by passivating the metal surface to achieve a reverse selective barrier of ALD TaN, with a reduced barrier at the bottom [297]. Figure 31 demonstrates the selective barrier integration flow in Cu interconnection; the via resistance could be reduced by 50% by using this new ALD TaN process, and the performance reliability (e.g., TDDB and EM) shows that no major difference was observed in the selective barrier scheme [297].

**Figure 32:**
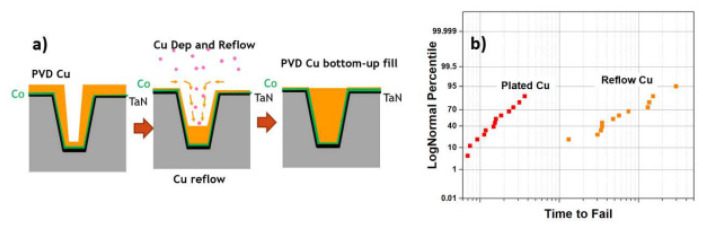
(**a**) Schematics showing Cu reflow process. (**b**) Electromigration lifetime values for plated Cu vs. reflow Cu with Co liner at 32 nm pitch [299]. Reprinted with permission from ref. [299]. Copyright 2021, IEEE Publisher.

Copper-reflow is a potential gap-fill solution as it eliminates the copper seed overhang formation and creates the perfect bottom-up fill to replace electrochemical plating (ECP). Beyond advanced 7 nm technology, Samsung adopted a “Cu reflow” process improving copper fill ability in smallest design features as part of Back End of Line (BEOL) contacts and metal islands, ensuring manufacturability by aggressive chip scaling [298]. Figure 32 shows the flow diagram for the Cu reflow process on Co liner and the corresponding improvement in electromigration (EM). The reflow process has better gap-fill (lower defectivity); therefore, it improves EM lifetime values [299]. Grain boundary diffusion is the main failure mechanism of EM and SM when a good copper line capping layer is used. Due to the use of higher process temperatures, the copper reflow process results in larger grain sizes of the deposited copper film compared to the copper electroplating process, thus exhibiting better SM and EM performance [298].

Another potential solution is to use other metals with good conductivity instead of copper conductors [300]. Daniel Gall evaluated five elemental metals (Co, Cu, Rh, Ru, Ir) with first-principle simulations and transport measurements on epitaxial layers. Rh and Ir are promising because their ρ_o_ × λ is the smallest (ρ_o_: the bulk resistivity, λ: the bulk electron mean free path). However, the probability of grain boundary reflection for Rh and Ir is approximately twice that of copper. Ru is considered a candidate for interconnection metal because of its ρ_o_ × λ value is 24% lower than copper. But more importantly, narrow Ru interconnected lines require a thinner barrier layer compared to copper interconnected lines. [301] Seong Jun Yoon et al. demonstrated that maximizing the grain sizes in Ru interconnected lines can effectively lower the total line resistivity [302]. The total line resistivity has been successfully reduced by more than 30% by suppressing the grain boundary scattering effect.

Some studies have proposed new integration schemes such as subtractive patterning flows as an alternative to dual damascene (DD) interconnection [299,303]. The subtractive patterning flow uses non-Cu conductors (Ru) that can enable lower R, better reliability, and potentially even provide 1x line R reduction (for line width < 12 nm) [303]. This method firstly forms the via layer in low k, and then fills the via and trench layers with metal deposition. The advantage of this process method is that it does not require metal–chemical mechanical polishing (CMP) and barrier layers. Then, the metal Ru is patterned using EUV single-exposure and subtractive etch to generate lines with CD down to 10.5 nm, which eliminates plasma damage to low-k trenches [304]. This method has good process control, stability, and a very high production line yield. These indicate that the subtractive etch of Ru is a feasible solution for advanced interconnected technology nodes.

An electroplating void-free cobalt interconnection was developed by Lam Research Tighe A. Spurlin [305]. Superconformal bottom-up Co deposition with a high aspect ratio was formed using a H^+^ and single suppressor at low current and long plating time. Compared with the copper/porous low-k SiOCH film integration process, the cobalt/porous low-k SiOCH film integration process exhibits less degradation in its electrical characteristics and reliability under thermal and electrical stress. The results indicate that the barrier free process can meet the requirements of Co interconnection, which is a promising solution for advanced interconnected technology nodes [306].

By introducing a via pre-filling process, a more significant change has been made to the conventional gap-filling process flow. This metalization process in the dual damascene structure is called a hybrid metalization. The hybrid metalization scheme of replacing metal pre-filled vias can reduce the aspect ratio of trench/via. This alleviates the poor Cu gap filling related to the scaling of the barrier/wetting layer, thereby increasing the Cu volume fraction in the trenches. A selective deposition process is applied in this scheme. By introducing the pre-filled Co process, seamless and bottom-up filling of metals was achieved in advanced interconnections [307,308]. The report demonstrated [308] a high selectivity CVD Co deposition has been achieved on copper, which can fill vias with a diameter of 45 nm and an aspect ratio of 3:1 in a copper dual damascene structure. IMEC and Lam have also demonstrated the feasibility of using the electroless deposition (ELD) technology to deposit Co as a pre-filled via material [307,309,310]. The main advantage of Co via the pre-filling process is that it can reduce via-layer resistance. As the via CD shrinks, compared to the conventional PVD-ECP via process, the chemical Co via process has a greater relative resistance reduction. For example, the via resistance of CD at about 40 nm is reduced by about 30% [309]. By using a hybrid Cu metalization with Co pre-filled via, the resistance in the 87° tapered vias can be reduced by 42% in the via of a 12 nm half pitch, and the resistance in the chamfered vias can be reduced by up to 52% [311]. The selective CVD Ru process is also used in the hybrid metalization scheme of Ru, which involves pre-filling Ru and then metalizing copper trenches [312]. The Ru prefill clearly decreased the via resistance for the Cu metalization in the 21 nm metal pitch. When using 1 nm TiN as the barrier layer with Co and Ru fully filled as the reference at this dimension, the resistance of the Ru–Cu hybrid metalization was reduced by 35%. The EM performance of Ru–Cu hybrid metalization is similar to the full copper metalization. A selective tungsten (W) deposition has also been used for via pre-filling [313]. Compared with the Cu dual damascene-filled via layer, the via resistance of W–Cu hybrid metalization is reduced by 40%. Therefore, the selective deposition hybrid metalization of contact and via pre-fill may be applied in the miniaturization process of future advanced interconnected technology nodes.

Intermetallic compounds (such as, Cu_2_Mg, AlNi, Al_3_Sc, AlCu, and Al_2_Cu) are proposed as candidate interconnected materials for advanced semiconductor devices [314,315,316,317]. The stoichiometric NiAl film with a thickness of 56 nm exhibits a resistivity of 13.9 µΩ cm after annealing at 600 °C. Additionally, different capping layers were tested to overcome the formation of metal surface oxides to achieve low resistivity [316]. The resistivity of 24 nm Al_3_Sc thin film can reach 12.5 µΩ cm after post-deposition annealing at 500 °C. At a thickness of 20 nm and above, the conductivity of AlCu and Al_2_Cu films is better than that of Ru films, and the resistance of AlCu and Al_2_Cu films with a thickness of 28 nm is only 9.5 µΩ cm [314]. Meanwhile, Al_2_Cu exhibits low resistivity, excellent gap-filling performance, and good reliability in TDDB, EM and BTS [315]. Based on the above series of good properties, Al_2_Cu may become an alternative to Cu [314]. Cu_2_Mg intermetallic compound shows a low resistivity of 25.5 μΩ cm and good gap filling performance by sputtering reflow. The thickness dependence of the resistivity of Cu_2_Mg thin films is better than that of Cu and Co, which is comparable to that of Ru. Cu_2_Mg is also considered an excellent interconnection material due to its excellent properties [317].

## 11. Advanced Devices Reliability

**Figure 33:**
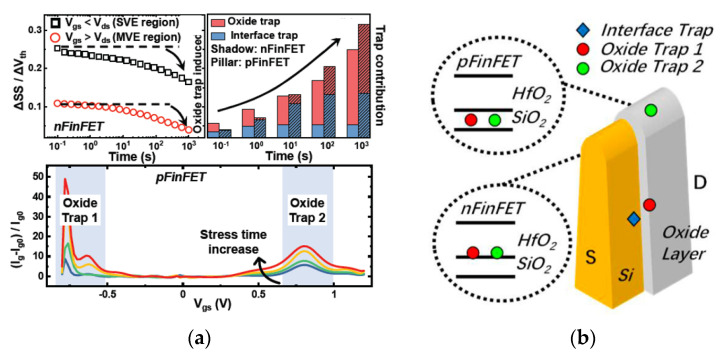
(**a**) Different oxide traps in FinFET illustrated in SILC spectrums, notated as oxide trap1 and oxide trap 2. (**b**) Typical positions of the interface traps as well as oxide traps created by HCD in a p- and n-type FinFET [321]. Reprinted with permission from ref. [321]. Copyright 2021, IEEE Publisher.

The reliability issues of advanced CMOS device beyond the 7 nm node are becoming more serious and complicated due to the 3D novel structure and nano-scaling [318,319,320,321], such as bias temperature instability (BTI), hot carrier degradation (HCD) and self-heating effect (SHE). Generally, trap-based approaches play very important roles in understanding the physical mechanisms in the study of reliability for advanced CMOS devices [321,322,323]. As we already know, there are interface traps in substrate/dielectrics and oxide trap in the dielectrics. Taking the 3D FinFET as an example, there are interface traps and two oxide traps (named trap 1 and trap 2) shown in Figure 33a [321]. Moreover, the locations of the interface trap and oxide trap 1 are crowded in the middle of the Fin side, while oxide trap 2 are in the Fin top in Figure 33b [321].

**Figure 34:**
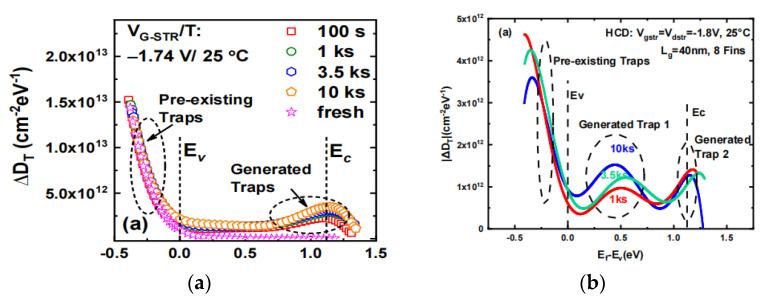
(**a**) Energy distribution of oxide traps in Si FinFETs for different NBTI stress time [323], reprinted with permission from ref. [323]. Copyright 2020, IEEE Publisher. (**b**) Energy distribution of oxide traps in Si FinFETs for different HCD stress time, and there are two generated oxide traps [324], reprinted with permission from ref. [324]. Copyright 2021, IEEE Publisher.

Moreover, to deeply understand oxide traps in terms of the reliability of the advanced CMOS device, the energy distribution of the oxide trap is also widely studied using the discharging-based multi-pulse (DMP) technique [322]. According to the origin of oxide traps, oxide traps can be classified as pre-existing oxide traps and generated traps, which are related to the process and stress, respectively. As shown in Figure 34, pre-existing oxide traps are above the valance band of Si in both (a) negative-bias-temperature instability (NBTI) stress and (b) HCD stress [323,324]. However, two generated traps are located at 0.4 eV above E_v_ and near the conduction band (E_c_) of Si, respectively (Figure 34). Therefore, in HCD degradation of FinFET, both locations and energy distributions of oxide traps show the two kinds of generated oxide traps.

**Figure 35:**
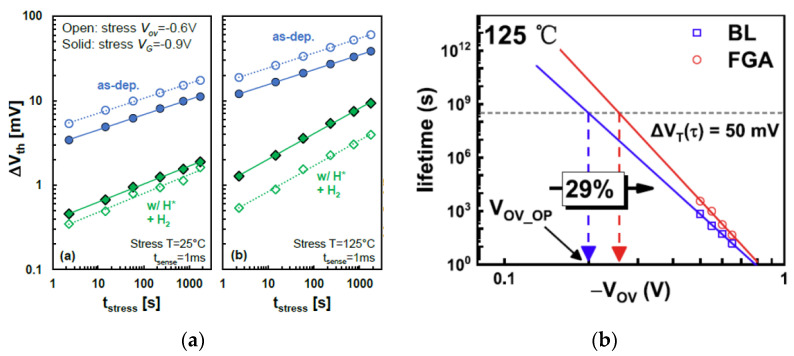
(**a**) NBTI-induced ΔV_th_ for different stress time measured at the low-T RMG pMOS processed w/o and w/H* − H_2_ IL treatment at 25 °C and 125 °C [325], reprinted with permission from ref. [325]. Copyright 2021, IEEE Publisher. (**b**) Comparison of extrapolated lifetimes between baseline transistors and FGA-optimized ones [326], reprinted from ref. [326]. Open Access, 2021, IEEE Publisher.

From the physical understanding of trap locations and trap energy distribution in the advanced CMOS device, the reliability of the HKMG stack process is optimized, especially for the annealing process. As shown in Figure 35a, a low-temperature atomic and molecular hydrogen annealing method is proposed to enhance chemical interfacial layer quality. Furthermore, the NBTI degradation is also clearly decreased by almost one order due to the passivation of the hydroxyl-E’ defects [325]. Figure 35b shows that optimized formation gas annealing (FGA) is an effective route to improve the 29% NBTI lifetime by reducing the generated oxide trap [326]. Therefore, in the study of the reliability of the advanced CMOS device, a trap-based approach is proven to be a useful and effective technique to improve reliability by process optimization. At the same time, metal thickness variation in the stacked nanosheet transistor will also affect the threshold voltage (V_th_) due to the changed wave function of metal gate, thus contributing to the significant V_th_ variability.

**Part Three: Materials in Beyond Moore Era**

## 12. III–V Materials

**Figure 36:**
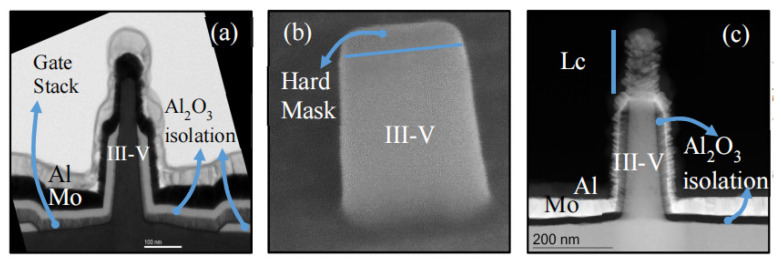
(**a**) TEM image showing across the width of single nanosheet FET; (**b**) SEM image of a single vertical nanosheet after dry etch; (**c**) TEM image of a vertical nanowire resistor after TLM process step [333]. Reprinted with permission from ref. [333]. Copyright 2017, IEEE Publisher.

High-mobility III–V materials used as channel s for GAAFET have been demonstrated as excellent candidates for high-speed and low-power circuits; these have been proposed for use in sub-10 nm CMOS technology [327,328]. Meanwhile, there is a large challenge to grow high-quality III–V layers on Si substrate for III–V GAAFETs, which stems from the incompatibility of III–V with the Si-based process [329,330]. Early studies on III–V GAAFET were focused only on simulation [331,332]. S. Ramesh et al. [333] presented high-performance devices of In_0.53_Ga_0.47_As vertical nanowire (VNW) and vertical nanosheet (VNS) by using dry etching. Figure 36 illustrates the TEM and SEM micrographs of In_0.53_Ga_0.47_As NS transistors and fabrication. The outcomes showed that scaling the effective oxide thickness together with (NH_4_)_2_S channel treatment and forming gas annealing improved the transistor characteristics, for example, Q (Gm/SS) by over 55%. In these transistors, a minimum SS = 63 mV/dec was obtained at V_DS_ = 0.5 V, and I_ON_ = 397 μA/μm at I_OFF_ = 100 nA/μm, while the G_m_ peak was at 1.6 mS/μm and maximum Q = 21. These results were the best values obtained so far for vertical III–V transistors.

However, a 1D nanowire can exhibit additional advantages for many purposes, e.g., higher stress relaxation, possibility of complicated gate stacking integration, more effective adsorption, and trapping of light [334]. X. Zhao et al. [335] reported In_0.53_Ga_0.47_As VNW MOSFETs of 7 nm in diameter. The transistors were processed by a top-down approach by applying RIE (reactive ion etching), alcohol-based digital etching, and Ni alloyed metal contacts. The research showed a record I_ON_ of 350 μA/μm at I_OFF_ = 100 μA/μm and V_DD_ = 0.5 V. The transistors showed a peak transconductance (gm, pk) of 1.7 mS/μm and minimal SS of 90 mV/dec at V_ds_ = 0.5 V, reaching the highest quality factor 19.

**Figure 37:**
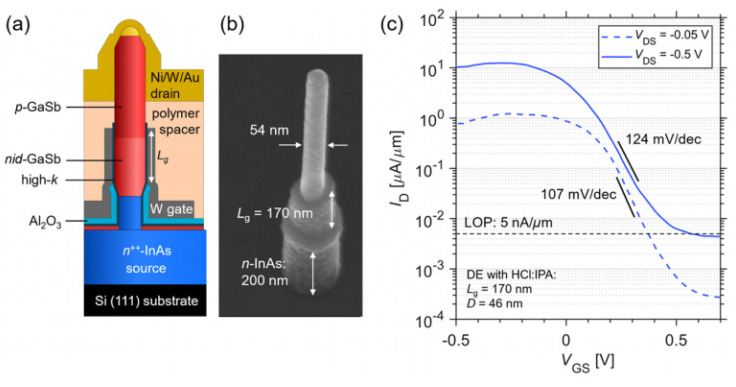
(**a**) Schematic image of a single NW MOSFET with GaSb channel where digital etching was used as the first step of the process; (**b**) SEM micrograph of a single NW transistor after defining the gate length; (**c**) transfer characteristics of the NW transistor with two-cycle DE- HCl:IPA 1:10 prior to high-κ deposition [336]. Reproduced from [336], open access by ACS, 2022.

Other types of III–V nanowires, e.g., InAs and GaSb, were also reported to be integrated for Si-GAAFETs. Among these studies, for example, Z. Zhu et al. [336] reported a vertical NW p-MOSFET with the GaSb channel by applying digital etch (DE) schemes. Figure 37a shows the schematic of a single NW-GAA MOSFET. In this study, two types of processes which are based on buffer-oxide etcher (BOE), 30:1 and HCl:IPA 1:10, are demonstrated and compared. Figure 37b shows the SEM image of a single NW transistor after gate length definition. By optimizing the DE conditions for GaSb NW MOSFETs, transistors with DE-HCl:IPA showed a minimum SS of around 107 mV/dec, while the I_ON_/I_OFF_ ratio could be increased by over three orders of magnitude (see Figure 36c). At present, high-mobility III–V channel materials instead of conventional Si is an important direction of advanced CMOS technology, and some research progress has been obtained. However, crystal quality optimization for Si-based III–V materials is still very challenging to achieve good device performance.

Si-based optoelectronic integration chips (OEICs) are a promising development in advanced CMOS technology. However, conventional IV-group materials of Si or Ge are indirect band structure, which result in low emission efficiency. In contrast, most group III–V materials have a direct bandgap, illustrating stronger efficiency of photon absorption and emission, nailing themselves for the optoelectronic devices in OEICs. To achieve a monolithic integration of III–V devices on the Si platform, it is important to develop a heteroepitaxy approach. There are some challenges for high-quality III–V heteroepitaxy on Si such as antiphase boundary (APB) and threading dislocation density (TDD) [329,330,331,332,333,334,335,336,337,338]. Y. Du et al. reported comprehensive research about the defect engineering methods for the growth of III–V materials on Si, which provided guidance for the optimization of high-quality III–V heteroepitaxy [329].

**Figure 38:**
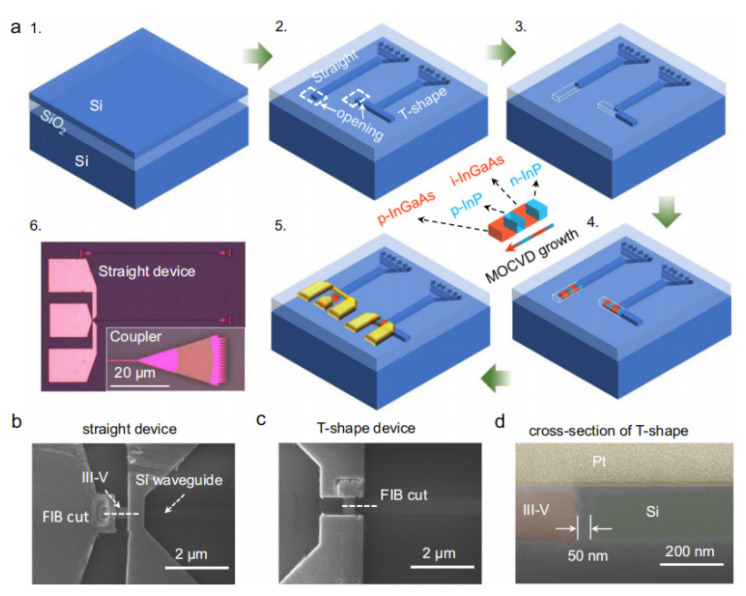
Illustration of process fabrication using template-assisted selective deposition. (**a**) Schematic of the process flow: 1. Preparation of SOI wafer. 2. Patterning of the Si top layer and openings. 3. Etch-back of Si to create hollow SiO_2_ template with a Si seed. 4. MOCVD of a p-i-n structure, the small arrow in the picture displays the growth direction. 5. Formed Ni/Au contacts. 6. Optical microscopy micrograph of a straight device and the coupler in the inset. (**b**) Top-view SEM micrograph of a straight device. (**c**) Top-view SEM image of a T-shape device displaying the FIB cut line. (**d**) Cross-section SEM micrograph of a T-shape device displaying an oxide-filled gap (with 50 nm width) separating the III–V-based active material and Si waveguide [342]. Reproduced from [342], open access by Springer Nature, 2022.

Even though high-epitaxy-quality III–V materials can be utilized for GAAFET or optoelectronic applications, the vertical geometry can be complicated in terms of device processing, and further engineering is necessary for on-chip integration as well as for waveguide-coupled solutions [339]. Template-assisted selective epitaxy (TASE) was considered as an effective method to overcome these limitations [340,341]. P. Wen et al. [342] demonstrated scaled and waveguide-coupled III–V photodiodes monolithically integrated on Si by TASE, implemented as InP/In_0.5_Ga_0.5_As/InP p-i-n heterostructures. Figure 38 illustrates the process of device fabrication on a conventional silicon-on-insulator (SOI) substrate using TASE. In Figure 38a, the dielectronic layer used as a template is patterned and drilled to expose the Si seed below. TMAH would then be used to etch the exposed Si and form the (111) face, following the epitaxy of III–V materials to fill the cavity. Since the facet is small and along the (111) direction, APBs and TDs generated at the (111) facet would be blocked by dielectronic template. As for device fabrication, an n-InP/i-InGaAs/p-InP/p-InGaAs multilayer structure was deposited within the template for metal–organic chemical vapor deposition (MOCVD). Two types of transistor architectures, namely “straight-shape” in Figure 38b and “T-shape” in Figure 38c, were compared. Figure 38d demonstrates the SEM cross-section image of a T-shape transistor which was cut by the focused ion beam (FIB): a small oxide-filled gap separated the Si waveguide from the vertically deposited III–V structure. This study showed a responsivity of 0.2 A/W at −2 V and a dark current of 0.048 A/cm^2^ at −1 V.

## 13. Substrate Engineering (GOI and GeSnOI)

GeSn has attracted attention due to its bandgap tunable and high-mobility properties [343,344,345]. Compared with Si and Ge, GeSn has a wide window of absorption coefficient in the short-wave infrared, and high carrier mobility. Compared with bulk GeSn/Ge materials, the GeSnOI substrate has higher mobility due to the removal of its high defect density Ge layer, as well as the following properties: higher operating temperature, greater optical confinement, higher light emission efficiency, higher net gain, resonator effect, low leakage current, lower coupling loss with waveguide, and greater suitability for integration of photonic devices with CMOS devices. It is a promising Si-based optoelectronic platform for optoelectronic devices and high-speed electronic devices materials. Therefore, we conclude this section by summarizing the growth of GeSnOI substrates, GeSnOI transistors, optoelectronic devices, and device simulation.

### 13.1. Growth of GeSnOI Substrates

Using patterned substrate and epitaxial overgrowth, Masashi Kurosawa et al. fabricated a GeSnOI substrate with 2% Sn. Square grooves of 10 × 10 μm^2^ were formed by wet etching of SiO_2_to make a seed layer window, and then Ge (50 nm), Sn (10 nm) and Ge (50 nm) thin films were sequentially deposited by vacuum evaporation system, followed by 800 nm SiO_2_. After rapid thermal annealing at 1000 °C for 1 s, Ge/Sn diffused and mixed to form GeSn material [346]. In 2019, Youki Wada et al. used a rapid fusion growth method, called “nucleation-controlled liquid phase crystallization” (NCLPC), to grow a GeSn layer on a quartz substrate by MBE, etch out GeSn lines by photolithography, and then cover with SiO_2_, Finally, slow cooling annealing is performed at a temperature higher than the melting temperature of GeSn. The resulting NCLPC-GeSn lines are almost intrinsic, and the charge carriers not only depend on the Sn concentration but also increase with the cooling rate in NCLPC [347].

In 2016, Dian Lei et al. reported that using MBE to fabricate GeSnOI substrates with Sn concentration of 4.0% and GeSn layer has −0.1%compressive strain. A 100 nm thick Ge_1-x_Sn_x_ layer was deposited on a Si at 180 °C. Later, a 500 nm-thick silicon dioxide layer was deposited on the Ge_1-x_Sn_x_ layer by using PECVD. The wafers were bonded by applying the direct wafer bonding (DWB) technique. The bonded wafers are then post-bond annealed in the N_2_ environment. Si epitaxial wafers are etched in TMAH solution [348].

In 2017, the fabrication method was improved. GeSnOI substrates were fabricated by epitaxial Ge on Si wafers, followed by CVD growth of Ge_0.92_Sn_0.08_ direct bonding and layer transfer methods. Among them, the Sn concentration measured by XRD (004) is 8.0%, and the Ge_0.92_Sn_0.08_ has −0.9% compressive strain [349,350,351,352].

In 2018, Krista R Khiangteet al. used MBE to grow Gd_2_O_3_ as an insulating layer on a 12-inch Si<111> substrate, increasing to 8% GeSn on Gd_2_O_3_ at 180 °C, and the compressive strain was −0.9% GeSnOI. The surface RMS roughness of Si<111> was 3.5 nm, almost twice that of the GeSnOI<001> substrate (~1.9 nm) fabricated by DWB [353].

In 2018, Tatsuro Maeda et al. fabricated GeSnOI substrates on the Ge<001> surface by direct bonding with a concentration of 6.95% and −1.0% compressive strain, respectively, with a thickness as low as 10 nm.

X. Wang et al. demonstrated how to form α-GeSn layer and finally formed a GeSnOI substrate by using PVD evaporation to deposit Ge and Sn on a Si wafer with 5 nm natural oxide through rapid thermal annealing [354].

In 2020, A. Elbaz et al. fabricated a microdisk cavity made of Ge_0.946_Sn_0.054_ alloy. The epitaxial GeSn layer of the Ge substrate is bonded on the Al substrate with a layer of SiN_x_ with compressive stress. A tensile strain of 1.4% was introduced in GeSn using all round SiN_x_ [355]. In 2021, D. Burt fabricated a GeSnOI substrate with 6% Sn content by using LPCVD epitaxy and direct bonding. The Al_2_O_3_/GeSn/Ge/Si epitaxial wafer is bonded to the Al_2_O_3_/SiO_2_/Si oxide wafer by direct bonding. The Si layer and the CMP Ge layer are etched with a KOH solution with a concentration of 30% and a temperature of 80 °C, and the GeSn disc is formed by etching to expose the Al_2_O_3_ layer. The above KOH solution is used to etch the Al_2_O_3_ layer again to obtain suspended structures and strain-relaxed GeSnOI substrate [356].

In 2022, Z. Kong et al. used an RPCVD epitaxial-grown Ge buffer layer and GeSn layer on the Si<100> substrate, and ALD Al_2_O_3_for fusion bonding with the oxidized Si substrate. Finally, the GeSnOI substrate with relatively less defect density was obtained by wet etching [357]. Table 4 summarizes some parameters of published GeSnOI substrates, Sn concentration, growth method of GeSn layer, bonding type, stress generated, and the crystal orientation of the wafer.

### 13.2. GeSnOI Transistor

In 2017, D. Lei et al. processed FinFETs on Ge_0.96_Sn_0.04_ with channel length and fin width of 50 nm and 20 nm, respectively. The measured SS values were smaller than 90 mV/decade. Meanwhile, the long channel transistors demonstrated a lowest SS of 79 mV/decade with a G_m_,int/S_sat_ of ~8.6. The results showed the morphology and thermal stability of the Ge_0.96_Sn_0.04_ fins on the GeSnOI substrate are remarkably different than blanket GeSn layers, while these Ge_0.96_Sn_0.04_ layers had better thermal stability [260,261,262,263,264,265,266,267,268,269,270,271,272,273,274,275,276,277,278,279,280,281,282,283,284,285,286,287,288,289,290,291,292,293,294,295,296,297,298,299,300,301,302,303,304,305,306,307,308,309,310,311,312,313,314,315,316,317,318,319,320,321,322,323,324,325,326,327,328,329,330,331,332,333,334,335,336,337,338,339,340,341,342,343,344,345,346,347,348,349,358,359,360,361,362,363,364,365,366,367].

In 2018, the National University of Singapore’s team studied the strain relaxation when a biaxially strained Ge_1-x_Sn_x_ layer processed to a Ge_1-x_Sn_x_ fins. In the beginning, the strain in the biaxially strain of compressively Ge_0.92_Sn_0.08_ layer is measured to be ~1%, and Raman spectroscopy measurements showed that strain relaxation is being increased with decreasing W_Fin_. The strain relaxation occurs only in the lateral direction, and a full relaxation can be obtained when the fin width is smaller than 30 nm [352].

**Figure 39:**
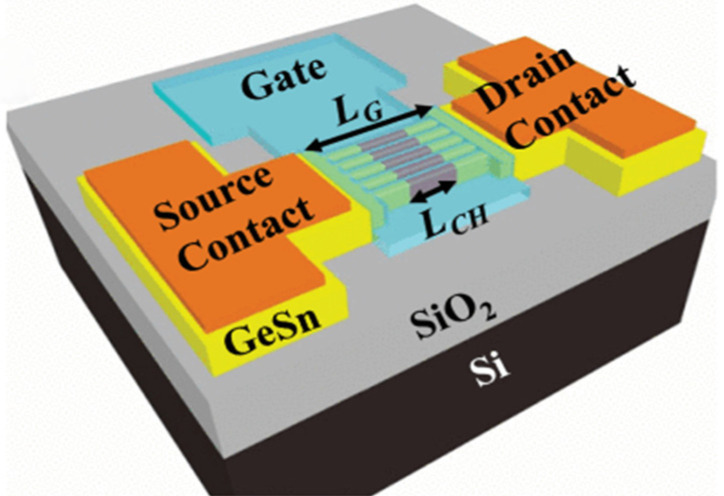
Three-dimensional schematic picture of a FinFET designed on GeSnOI wafer with multiple parallel fins [354]. Reproduced from [354], open access by OSA, 2018.

In 2018, D. Lei et al. reported a study about the effect of post-metal annealing (PMA) on the electrical performance of p-channel GeSn FinFETs (Figure 39). The transistors showed a high mobility of 295 cm^2^V^−1^s^−1^ at a reverse carrier density N_inv_ of 8 × 10^12^ cm^2^ V^−1^s^−1^ after 400 °C PMA. Further investigations showed the SS improved from 153 mV/decade to 139 mV/decade for transistors with gate length of 4 μm and gate width of 40 nm after PMA treatment. The Dit value decreased from 1.19 × 10^13^ cm^−2^eV^−1^ to 5.8 × 10^12^ cm^−2^eV^−1^ after 400 °C PMA [368].

D. Lei et al. p-FinFETs fabricated by optimized etching. When V_DS_ of −0.05 V, SS is 83 mV/decade, and the I_ON_/I_OFF_ ratio exceeds four orders of magnitude. At a gate bias (VG) of −1 V, the gate leakage current (I_G_) is maintained at ~2 × 10^−4^ μA/μm. When V_DS_ is −0.05 V, the GeSn p-FinFET device has a minimum S value of 79 mV/decade when W_Fin_ is 30 nm and L_CH_ is 200 nm [354].

Later, D. Lei et al. reported p-FinFET on Ge_0.95_Sn_0.05_OI with a fin width of sub-10 nm, while the top widths were as small as 5 nm. The transistors with a channel length of 50 nm showed a minimum SS value of 63 mV/decade and a maximum Gm, int 900 µS/µm (V_DS_ of −0.5 V) [369].

**Figure 40:**
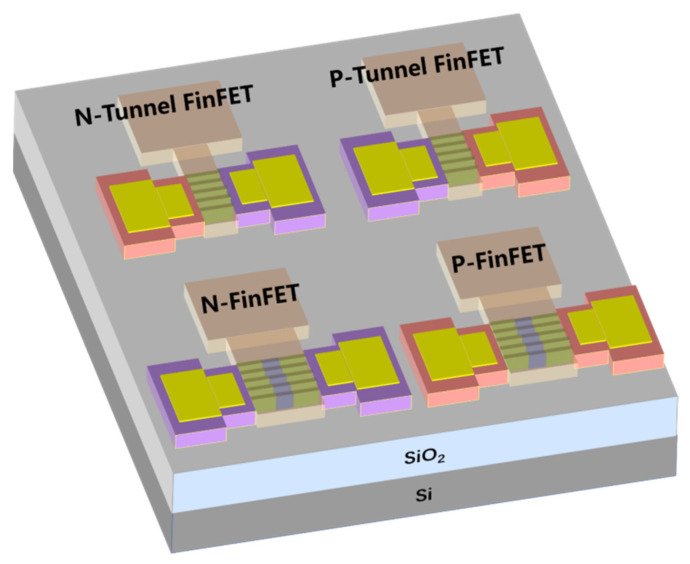
Three-dimensional schematic design of the integrated FETs on GeSnOI substrate [370].

K. Han et al. demonstrated both GeSn n- and p-channel FinFETs and TFFETs on the same substrate for the first-time integration (Figure 40). GeSn p- and n-FinFETs with fin width of 20 nm showed SS values of 110 and 120 mV/decade, respectively. Meanwhile, by applying a bias voltage of 1.5 V, SS was as low as 20 mV/decade for both n- and p-TFETs [370].

Yuye Kang et al. reported a GeSn GAA p-FET formed on GeSnOI substrate. The transistors in this study had a channel length of 60 nm and a gate width of 15 nm where a good SS value of 74 mV/decade was obtained [371]. Later, Shengqiang Xu et al. reported the fabrication of a GeSn p-FinFET with a channel length and fin width of 80 nm and 40 nm, respectively. This study demonstrated a SS value of 114 mV/decade while I_ON_/I_OFF_ ratio was increased about 4 orders of magnitude in the linear region [372].

In 2021, Yuye Kang et al. showed Ge_0.95_Sn_0.05_ p-GAAFET with an NW width of sub-3 nm and L_CH_ of 60 nm on Ge_0.95_Sn_0.05_OI substrate. The transistors demonstrated a SS value of 66 mV/decade, an I_ON_/I_OFF_ ratio of ∼1.2 × 10^6^, while the effective hole mobility (μ_eff_) was ∼115 cm^2^V^−1^s^−1^ [373].

In 2021, Guangyang Lin et al. optimized the anisotropic etching of Ge_0.875_Sn_0.125_ by inductively coupled plasma (ICP), and obtained a sidewall angle of 89° using Cl_2_ 100sccm, Ar_2_ 5sccm, O_2_ 10sccm, and P10mTorr. An SS value of 240 mV/dec for a fabricated 45 nm thickness Ge_0.875_Sn_0.125_OI back-gate transistor [374]. Table 5 summarizes the transistors fabricated with GeSnOI, such as substrate, transistor type, gate length, gate width, subthreshold slope, and I_ON_/I_OFF_ ratio.

Two references for GeSn lasers [375,376]: In 2021, Hyo-Jun Joo et al. fabricated a 1D photonic crystal (PC) nanobeam laser. The strain-free nanobeam laser demonstrated threshold density at 18.2 kW cm^−2^ for 4 K, which is remarkably lower compared to unreleased nanobeam one showing 38.4 kW cm^−2^ at 4 K [375].

A. Bjelajacet al. developed a Ge_0.831_Sn_0.169_OI at a compressive strain of −0.5% by bonding a thick layer of GeSn with partial strain relaxation. A room-temperature optically pumped operation GeSn laser was created in a microdisk resonator fabricated on the platform [376].

In 2017, Jadavpur University team used SILVACOATLAS to conduct a detailed study of numerous transistor parameters for simulation and logic circuit applications of strained GeSnOI MOSFETs with different Sn contents. Compared with the equivalent Ge transistors formed on GeOI (10 nm thick channel and 20 nm long), the Ge_0.94_Sn_0.06_ MOSFET showed an improvement of the peak transconductance gm, and peak gain Av of 80.5%, and 18.8%, respectively, as well as an improvement of peak cut-off frequency (f_T_) and maximum frequency of oscillations (f_max_) 83.5% and 81.7%, respectively. In addition, such transistors showed 78.8% improvement in I_ON_ and a 44.5% decrease in delay in comparison with Ge transistors [351].

In 2019, Jayanti Paul et al. of the University of Calcutta used TCAD to simulate a subthreshold model of a GeSn-on-insulator (GeSnOI) MOSFET based on a two-dimensional surface potential, which takes interfacial trapping and fixed oxide charge density as well as quantum effects into account. A 2D analytical model based on quantum effects was introduced to calculate different transistor parameters of GeSnOI pMOSFETs which relates to SCEs with channel lengths of 14 nm, channel thicknesses in range of 5–10 nm, while interface trapped charge densities within 10^12^ to 10^13^ eV^−1^ cm^−2^ and Sn composition in the interval of 0–6%. The GeSnOI MOSFET with 5 nm channel thickness demonstrated the lowest V_th_, DIBL, SS and V_th_ offset values. Furthermore, DIBL and SS increased slightly with Sn concentrations in the range of 0–6% [377].

## 14. Beyond Moore Era-Si Optoelectronics

With the rapid technological development in the information age, more and more demands for information transmission capability, computing capacity, and processing speed in the integrated circuit industry are required. However, there are various signs that “Moore’s Law” once followed by Si-based chips is declining. The development of Si-based integrated circuits has encountered many bottlenecks.

The field of optoelectronics has developed rapidly in recent years and has become one of the key technologies in next-generation data interconnection and communication systems [378]. People have been expecting to combine photonics and electronics processing to realize the Optoelectronic Integrated Circuit (OEIC). The OEIC can provide monolithic solutions with lower cost, higher performance, and higher integration density [379]. The widespread application of Si-based OEIC lies in the need to use CMOS-compatible fabrication processes to enable low-cost mass production, which is a key factor in introducing optoelectronics into a range of technical fields [380].

The minimum optical loss window of silica fiber Is in the wavelength range of 1300–1550 nm, and most long-distance data transmission also works in this window. Therefore, many photonic devices, e.g., lasers, detectors, and modulators, operating within this wavelength range can be directly integrated to external servers without wavelength conversion [381,382,383].

### Optoelectronic Integration

The integration of electronics with photonics provides more complicated optical systems and improves the performance of photonic ICs. There are two main types of integration: monolithic and multi-chip.

Monolithic integration can be realized by adjusting the CMOS processes to high-speed optics, or by placing the building optical circuits (for example by wafer bonding) within the steps of today’s CMOS flow. The latter method is more conducive to reducing costs, but it still faces some limitations for the development of high-speed devices.

**Figure 41:**
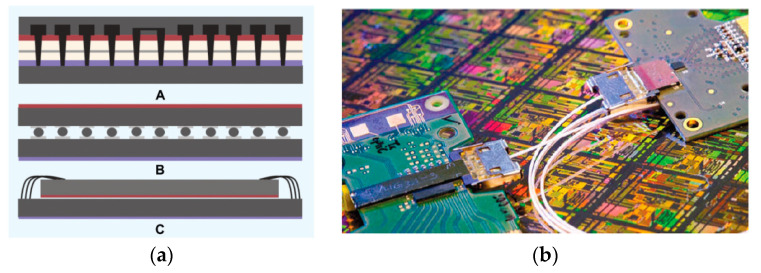
(**a**) A scheme for electronic and photonic integration: (A) through Si vias, (B) bump bonding, (C) wire bonding [385]. Reproduced from [385], open access by Walter de Gruyter, 2014. (**b**) Intel’s integrated link contains fully integrated Si photonic transmitter chip with hybrid Si lasers (**left side**) and a fully integrated receiver chip based on Ge photodetectors (**right side**) [386]. Reprinted with permission from ref. [386]. Copyright 2010, Springer Nature Publisher.

Multi-chip integration is based on two chips to be separately fabricated and later bonded together. Compared to the high cost of developing a monolithic integration process, this approach also has significant advantages to some extent. Compared to expensive electronic processes that require smaller critical dimensions, cheaper processes can be used to fabricate photonic devices, which significantly reduces manufacturing costs in total. A major challenge with the multi-chip integration method is necessary to obtain the high-speed connections between the processed chips. The electrical contact is carried out by bonding techniques for multi-chip integration, e.g., wire bonding, and flip-chip bump bonding, which decreases parasitic capacitance and higher density, can be obtained compared to wire bonding, using Si vias (provides even higher density and lower parasitics) (see Figure 41) [384,385,386].

**Part Four: Metrology technologies**

## 15. Advanced Material and Structural Analysis of Miniaturized CMOS

The scaling technology is approaching its physical limits where cost and reliability issues far outweigh the benefits. The requirements for the development of metrology are increasingly crucial in the semiconductor industry. Metrology enables control of the manufacturing process, helps production, reduce costs, and regulates the time-to-market for new products.

Over the past several years, complex 3D device structures, like FinFET and GAAFET, have been developed in CMOS technologies and beyond. New materials, patterning techniques and processes are also introduced to the fabrication of new generation devices. These all have been raising considerable challenges for all areas of metrology. For example, the challenges in measurement of the FinFET structure are amplified by shrinking dimensions [1,3]. There are difficulties in the analysis of Van der Waal bonded 2D materials such as graphene and other graphene-like materials [2,3]. The patterning techniques contain nanoimprint, extreme ultra-violet (EUV) lithography, and multi-patterning. These techniques will bring new challenges or difficulties for measuring critical dimensions (CD), defectivity and overlay [387,388,389].

In this section, we will discuss the recent progresses in metrology techniques.

### 15.1. Scanning Electron Microscopy (SEM)

SEM has been providing at-line or in-line imaging for analysis of in top or cross-sectional view of samples, particles, detecting defects and CD measurements. At this stage, more improvements are now demanded for effectively estimate CD and defect review at and beyond 10 nm node technology [390]. Ultra-low/high energy electron beams have been applied in SEM technique to overcome image degradation. By reducing the spherical aberration, the resolution of SEM can be improved. However, a few focus steps might be needed because the reduction in spherical aberration results in a small depth of field. SEM has also been used to construct the 3D structure features by using the multiple SEM beams [391,392] and detecting the backscattered electrons from multiple primary beam energies. This method is much faster than that of TEM techniques.

### 15.2. Transmission/Scanning Transmission Electron Microscopy (TEM/STEM) Technology

The aberration corrected lens technology is now commercially available, which can significantly improve the TEM/STEM technique’s resolution. Aberration-corrected STEM systems have been reported to image single layer graphene with defects in the stacking configuration of multilayer graphene [393,394].

### 15.3. X-ray Metrology

Mainstream X-ray metrology, such as HR-XRD [395,396,397,398], X-ray fluorescence (XRF), total reflection X-ray fluorescence (TXRF), XRR, X-ray photoelectron spectroscopy (XPS), and small-angle X-ray scattering (SAXS), has been used to determine thin film’s parameters, like thickness, composition, strain/relaxation state, tilt, and lattice constant.

It should be noted that SAXS is a technique routinely applied to study structural features with sizes of 1–100 nm. The technique can be used to measure any complex periodic nanostructures such as FinFETs in any dimension. Two types of SAXS techniques have been developed: transmission-based SAXS (or CD-SAXS) and grazing incidence SAXS (GISAXS).

CD-SAXS can analyze the features of sub-5 nm devices, e.g., 3D FinFETs. Since CD-SAXS is a transmission measurement through the substrate, then a high brightness light source is needed to obtain a decent throughput with a small spot size. Meanwhile, to use such strong light sources make this technique impractical in both laboratory and factory environments. Therefore, the improvement of sources in SAXS is of great challenge to realize the measurements of sub-5 nm devices.

While GISAXS has a larger spot size in comparison with CD-SAXS. The measurement time is remarkably shorter than CD-SAXS. This technique is generally applied to estimate the average size of nanoparticles or diameter of nanowires, pitch, height of Si gratings, and sidewall angle [395].

### 15.4. Raman Spectroscopy

Raman spectroscopy can measure stress. Recent research on SiGe devices demonstrated that in-line Raman spectroscopy is a fast and accurate measurement of composition and strain state 12.

**Figure 42:**
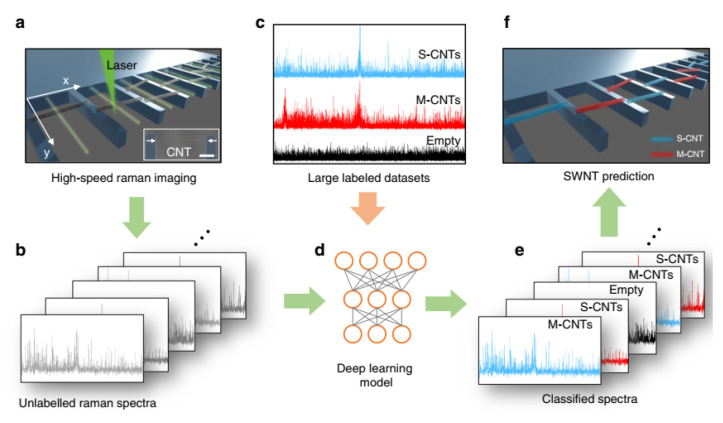
Schematic drawing of carbon nano tube (CNT) analysis using deep learning-based Raman spectra measurement. (**a**) Employment of high-speed Raman to image a fork-like sample. (**b**) Piling up and finally generating an unlabeled Raman spectrum. (**c**) To classify the large, labeled datasets into the following categories: S-CNTs, MCNTs, and empty. (**d**) Using the deep learning model. (**e**) Organizing of individual spectra applying the model. (**f**) Identifying the CNTs [399]. Reproduced from [399], open access by Springer Nature, 2022.

Moreover, by combining with the machine learning technique, the scanning rates were significantly increased. This in turn reduces the laser exposure time, which helps minimizing the laser-induced sample damage. This metrology has been demonstrated by measuring the features of suspended CNTs [399], as shown in Figure 42.

### 15.5. Hybrid Metrology

**Figure 43:**
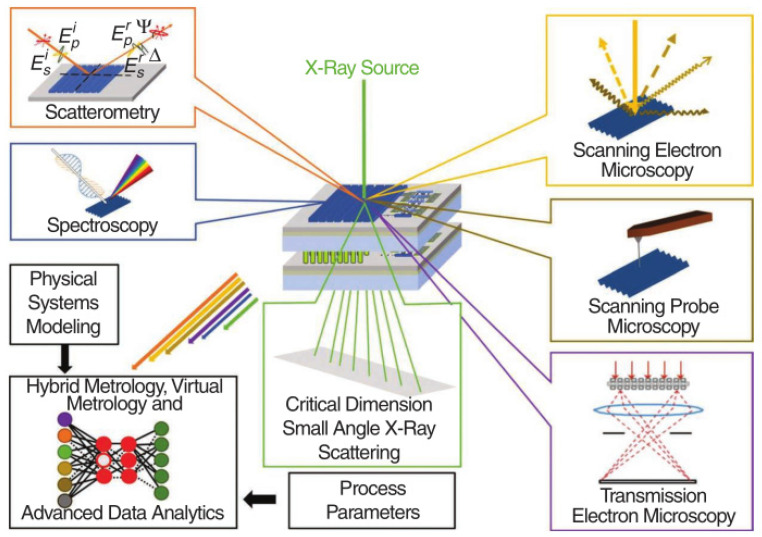
Multiple-techniques approach to device metrology [405]. Reprinted with permission from ref. [405]. Copyright 2024, IEEE Publisher.

Figure 43 shows a series of characterization methods for nanometer scale material. In general, Hybrid metrology has shown promising advantages in reducing the uncertainties in the characterization of sub-10 nm dimensions [400]. With the assistance of modelling data analysis and treatment algorithms, a set of parameters can be obtained from correlative tools by comparation and merge.

It has been reported that multiple patterning errors can be easily obtained by combining CD-SAXS with optical metrology [401]. The idea behind this is that the buried layers are usually opaque to optical metrology techniques which can simply be analyzed by CD-SAXS. By incorporating SAXS data into the optical models, an enormous improvement in the optical measurements can be obtained. Hybrid STEM systems equipped with EELS and X-ray detection are applied to obtain valuable analysis into interface chemical bonding.

As another example, AFM and TEM imaging tools have recently been combined for advanced 3D imaging. This combined metrology provides nm-resolved 3D electrical and chemical analysis of Si- and Ge-based structures for sub-20 nm technology nodes [402]. It is extremely effective when performing failure analysis, because multiple material properties can be acquired by using the entire spectrum of different AFM modes [403,404,405].

## 16. Conclusions

This review article is presented in four parts with the following outcomes:

In part one, GAAFET was presented as an excellent candidate for the 5 nm node technology and beyond due to its high control of SCEs. The design of GAAFET in vertical (vGAAFET) and horizontal (hGAAFET) was defined. One of the main advantages of vGAAFETs over hGAAFETs is the high integration density since the architecture limitations for gate length, spacer width, and S/D contact region for hGAAFET. For vGAAFET design a multilayer of SiGe/Si can firstly be grown and later, this original structure is patterned and vertically etched to form mesa structure. In vGAAFET design, SiGe channel is formed by selective etch in lateral direction.

One of the purposes of MOSFET size shrinking is also for the power consumption reduction. In general, reduced V_T_ and V_DD_ are eligible for lower power consumption. Other transistors issue refers to smaller SS requirement, where extremely steep switch characteristic is sought. As transistors have constant SS, reducing V_T_ and V_DD_ results in an exponential increase in leakage current and static power consumption. In the nano transistors, the SS of transistors has a limitation of ~60 mV/dec due to the tail of Boltzmann distribution. Therefore, a new design as such as TFET design which is based on the BTBT mechanism is presented to overcome the 60 mV/dec limitation of SS and scale further V_T_ and VDD with low leakage current. In this way, TFET is considered as excellent candidate for low power applications.

FDSOI technology was also presented as another approach to control SCEs during scaling down the transistor dimensions. The cost of the FDSOI wafer is higher, but it demands minor changes in production compared with normal bulk processing, giving more possibility to employ new technologies. If we compare the total cost for FDSOI transistors to GAA transistors, it is almost compatible. Therefore, due to the advantages of FDSOI and FinFET, as well as strain engineering, a new platform is being offered in the technology roadmap such as FDSOI- (or strained) FDsSOI-FinFETs. The effect of SiGe channel and doubly delta-doped layers on high frequency performance still need further study [406,407].

Transistor simulation using TCAD device models has also been discussed. As transistor devices scale down to the 5 nm technology node and beyond, a quantum-physics-induced effect is highly expected. This results in a huge rise not only in TCAD theory and methodology complexity but also in computational cost. To improve the predictability and computational efficiency of TCAD simulations, advanced physical models beyond drift-diffusion frameworks, as well as incorporating high-performance computing, have been presented. Further improvement can be achieved by introducing AI technology, including machine learning and deep learning approaches into TCAD and implemented not only atomistic calculation but also device- and circuit-level simulation.

In part two, advanced lithography techniques using self-aligned double pattern (SADP) and self-aligned quadruple pattern (SAQP) technology for 5 nm technology node and beyond were presented.

As the continuous shrinkage of transistor design rules, overlay requirement becomes more demanding. The mass production for advanced node requires overlay performance to be 2 nm and below in EUV platform. This is a real challenge for both overlay correction by scanner as well as overlay measurement. For the global overlay measurement, image-based overlay strategy has been mostly applied in mature products. As the technology node continues to shrink, diffraction-based overlay strategy has showed impressive performance. In this field, image-based overlay is better for non-tool-induced shift, whereas diffraction-based overlay is better for tool-induced shift.

Epitaxy growth of GeSi/Ge or SiGe/Si stacks where Si, Ge, or SiGe can be used as channel material of the GAAFETs have also been presented. Later, the Si or SiGe channel is formed by partial removal using wet etching.

For doping of GAAFET structures, the plasma doping method has been applied instead of traditional ion implantation.

For 3D CMOS, Ti silicide contact has been demonstrated as a suitable metal contact due to its low contact resistivity and high thermal stability. For such integration, pre-amorphous Ge implantation is performed to improve the property of Ti silicide.

HfO_2_ as high-k material has thermal instability at HfO_2_/Si interface in nanoscale transistors. A solution for this problem is introducing a SiO_x_ interlayer between HfO_2_ and Si substrate. Meanwhile in each technology node, the thicknesses of SiO_x_ and HfO_2_ have constantly decreased.

TiN is used as PMOS work function metal and a TiAl-based alloy is used as the NMOS work function metal.

For the latest scaling down in technology nodes, copper interconnection shows difficulty in maintaining the trade-off between line resistance and reliability.

One solution is forming a barrier which is an alloy of Cu with V, Al, or -Mn seed layer by post-metalization annealing.

The reliability issues of an advanced CMOS device beyond the 5 nm node remains a complicated issue due to bias temperature instability, hot carrier degradation, and the self-heating effect. Usually, the traps are involved in physical mechanisms in reliability studies for advanced CMOS devices. Based on the physical understanding of location and energy distribution of traps in CMOS devices, the reliability of the process of HKMG stack in CMOS devices is optimized, especially the annealing process.

In part three, the material technology for sub-10 nm GAAFET and beyond the Moore era has been discussed. Later, the content covers the integration of III–V materials as high-mobility channel application. The challenges of the high-quality growth of the III–V layer on the Si substrate and the difficulties in manufacturing III–V GAAFET were highlighted.

The application of 1D nanowire of III–V for transistor application was highlighted. The benefits of nanowire integration are due to the capability of advanced gate stacking, better stress relaxation, and more effective light trapping and adsorption. InAs, GaSb nanowires were presented for use in manufacturing Si-GAAFET devices.

Then, Si-based OEICs were proffered as a promising development in advanced CMOS technology. Most group III–V materials have direct bandgap, illustrating stronger efficiency of photon absorption and emission, nailing themselves for the optoelectronic devices in OEICs. To achieve a monolithic integration of III–V devices on the Si platform, it is important to develop a heteroepitaxy approach. There are some challenges for high-quality III–V heteroepitaxy on Si such as antiphase boundary and threading dislocation density.

Later, GeSn material was introduced due to its tunable bandgap and high-mobility properties. Compared with Si and Ge, GeSn has a wide window of absorption coefficient in the short-wave infrared, and higher carrier mobility. Compared with bulk GeSn/Ge materials, GeSnOI substrate has higher mobility due to its’ high defect density Ge layer been removed. More discussions about the manufacturing and using of GeSnOI substrates for optoelectronics application was presented.

In part four, metrology technologies were discussed. As the scaling technology is approaching its physical limits, the requirements for the metrology development becomes increasingly crucial in the semiconductor industry. Metrology enables manufacturing process control, helps production, and reduces costs and the time-to-market for new products.

The aberration-corrected lens technology is adopted to improve the TEM/STEM technique’s resolution. Mainstream X-ray metrology, such as HR-XRD, XRF, TXRF, XRR, XPS, and SAXS, has been used to determine thin film’s parameters, like thickness, composition, strain/relaxation state, tilt, lattice constant.

It should be noted that SAXS is a technique routinely employed to study the structural features with the sizes ranging from 1 to 100 nm. The technique can be used to analyze any complex periodic nanostructures such as FinFETs in any dimension. The CD-SAXS technique can provide analysis of features and devices down to sub-5 nm, e.g., 3D FinFETs. CD-SAXS can perform a transmission through wafer measurement.

Hybrid metrology has demonstrated many advantages in the reduction in uncertainties for characterizing sub-10 nm dimensions. As an example, hybrid STEM systems equipped with EELS and X-ray detection are applied to provide valuable analysis into interface chemical bonding.

## Figures and Tables

**Table 1:** Some experimental works on TFETs based on Si and Ge materials.

Year	Ref.	Material System	I_ON_ [μA/μm]	I_ON_/I_OFF_ Ratio	SS_min_ [mV/dec]	<60?
2007	[38]	Si	12.1	10^4^	~52.8	√
2008	[39]	Si	<0.1	<10^7^	42	√
2009	[40]	Ge/Si	0.4	>10^6^	35	√
2010	[41]	Si	~2	10^8^	46	√
2011	[42]	Si	<10^−4^	10^4^	30	√
2012	[43]	Strain-Si	0.1	10^7^	76	×
2012	[44]	Si	~0.1	10^6^	52	√
2012	[45]	Si	0.15	~10^5^	36	√
2013	[46]	Ge/Si		10^7^	49	√
2014	[47]	Si	39	>10^5^	69	
2014	[48]	Si	20	10^8^	29	√
2015	[49]	Ge/strain-SOI	<1	10^7^	29	√
2016	[50]	Si	<0.01	10^5^	34	√
2017	[51]	Si	2.0	10^8^	75	×
2017	[52]	Ge/Si	14	10^7^	>200	×
2019	[53]	Si	<0.01	10^4^	17	√
2020	[54]	Si	40	~10^8^	69	×
2020	[23]	SiGe	<0.01	~10^6^	65.4	×

“√” or “×” stands for the SS is smaller or higher than 60 mV/dec.

**Table 2:** Some experimental works on TFETs based on III–V materials.

Year	Ref.	Material System	I_ON_ [μA/μm]	I_ON_/I_OFF_ Ratio	SS_min_ [mV/dec]	<60?
2011	[24]	Hetero-InGaAs	10	10^5^	55~60	√
2012	[25]	GaAsSb/InGaAs	135	10^4^	100	×
2012	[26]	InAs/Si	2.4	10^6^	150	×
2012	[27]	InAs/GaSb	180	10^4^	200	×
2012	[28]	InAs/Si	<1	~10^6^	21	√
2013	[29]	InGaAs/GaAsSb	740	10^2^	NA	×
2015	[30]	InGaAs	1	10^6^	64	×
2015	[31]	InGaAs/GaAsSb	84	<10^5^	64	×
2015	[32]	GaAsSb/InGaAs	275	10^5^	55	√
2016	[33]	InAs/Si	4		70	×
2017	[34]	Ge/GeSn	2.4	10^4^	215	×
2018	[35]	InGaAsSb/InAs	<10	<10^5^	75	×
2018	[36]	InGaAsSb/InAs	<0.1	<10^4^	35	√
2019	[37]	GaSb/InAs		<10^4^	40	√
2021	[22]	InGaAs/GaAsSb	<0.1	~10^3^	42	√

“√” or “×” stands for the SS is smaller or higher than 60 mV/dec.

**Table 3:** Summary of Ge (Sn) GAA NW FETs in terms of year, institution, Sn composition, channel length, I_ON_, I_ON_/_OFF_, SS, and stack number.

Year	Institution	Sn	Channel Length	I_OFF_ (μA/μm)	I_ON_/I_OFF_ Ratio	SS (mV/dec)	Stacks	Ref.
2017	National Taiwan University	6%	150 nm	1400	——	84	1–2	[189]
		10%	60 nm	1850	——			
2019	National Taiwan University	9%	——	120	——	84	3	[190]
2020	PGI 9	8%	150 nm	——	3 × 10^6^	72	1	[191]
2020	PGI 9	8%	——	——	3 × 10^6^	67	1	[192]
2021	National Taiwan University	10%	80 nm	86	——	——	4	[193]

**Table 4:** Summary of parameters of GeSnOI substrate, Sn concentration, growth method of GeSn layer, bonding type, stress generated, and crystal orientation of wafer.

Year	Sn Composition	GeSn Growth	Bonding Type	Strain	Direction of Wafer	Ref.
2012	0–3%	vacuum evaporation system	Seed epitaxy	3%	<100>	[346]
2017	8%	CVD	DWB	−0.9%		[349]
2018	8%	MBE	Epitaxy	−0.9%	<111>	[353]
2020	5.4%			1.4%		[355]
2022	7%	RP-CVD	fusion bonding	−0.32–0.47%	<100>	[357]

**Table 5:** The transistors fabricated with GeSnOI such as substrate, transistor type, gate length, gate width, subthreshold slope, and switching ratio.

Year	Transistor Type	Gate Length/nm	Fin Width/nm	Subthreshold Slope/mV/Decade	I_ON_/I_OFF_ Ratio	Ref.
2017	p-FinFET	50	20	<90	>10^4^	[350]
2018	p-FinFET	4 µm	40	139		[368]
2018	p-FinFET	200	30	79	>10^4^	[354]
2018	p-FinFET	50	5	63	10^4^	[369]
2018	p-FinFET	100	15	93	>10^4^	[354]
2019	p-GAAFET	60	15	74		[371]
2019	p-FinFET	80	40	114	10^4^	[372]
2021	p-GAAFET	60	3	66	1.2 × 10^6^	[373]

## Data Availability

Data are contained within the article.
